# Tight regulation of Earth’s long-term temperature over Phanerozoic time

**DOI:** 10.1038/s41467-026-72672-6

**Published:** 2026-05-04

**Authors:** Dongyu Zheng, Alex G. Lipp, Alexander Farnsworth, Shufeng Li, Andrew S. Merdith, Khushboo Gurung, Mingcai Hou, Anqing Chen, Zixi Hou, Daniel J. Lunt, Erik A. Sperling, Paul J. Valdes, Benjamin J. W. Mills

**Affiliations:** 1 State Key Laboratory of Oil and Gas Reservoir Geology and Exploitation & Institute of Sedimentary Geology, Chengdu University of Technology, Chengdu, China; 2 School of Earth and Environment, University of Leeds, Leeds, UK; 3 Department of Earth Sciences, University College London, London, UK; 4 School of Geographical Sciences and Cabot Institute for the Environment, University of Bristol, Bristol, UK; 5 State Key Laboratory of Tibetan Plateau Earth System, Environment and Resources (TPESER), Institute of Tibetan Plateau Research, Chinese Academy of Sciences, Beijing, China; 6 CAS Key Laboratory of Tropical Forest Ecology, Xishuangbanna Tropical Botanical Garden, Chinese Academy of Sciences, Mengla, China; 7 School of Physics, Chemistry and Earth Sciences, University of Adelaide, Adelaide, SA Australia; 8 Department of Earth and Planetary Sciences, Stanford University, Stanford, CA USA

**Keywords:** Palaeoclimate, Stratigraphy

## Abstract

Knowing the past temperature of the Earth is crucial for understanding the mechanisms driving climate change and biosphere evolution, but there is significant debate about the range of past temperature variation. Previous interpretations, largely based on oxygen isotope records, suggest that global temperature has generally declined over the past 539 million years, but substantial uncertainties persist. In this study, we introduce an independent estimate for long-term Phanerozoic temperature trends based on a large database of chemical weathering indices from siliciclastic sedimentary rocks, globally upscaled using a state-of-the-art general circulation paleoclimate model. Our results imply that Phanerozoic global temperatures remained within 10-30 °C, and that Paleozoic oceans had comparable temperatures to Mesozoic and Cenozoic oceans, in contrast to previous work suggesting that they were anomalously hot. This finding supports the idea that negative feedback processes, such as silicate weathering, have maintained long-term global average temperatures within a relatively tight range, contributing to the continued long-term evolution of the biosphere.

## Abstract (web summary)

This study reconstructs Earth’s temperature over the past 539 million years by integrating rock chemistry and climate models, showing that global temperatures remained within ~10–30 °C, challenging ideas of anomalously high early Paleozoic climates.

## Introduction

The Phanerozoic Eon, encompassing the past 539 million years of Earth’s history, saw an explosion of life in the oceans and on the land. This included the rapid expansion of multicellular organisms in the Cambrian–Ordovician^1^, the colonization of land by plants in the Ordovician-Devonian^2^, and the appearance and rise to dominance of mammals in the Triassic–Paleogene^3,4^. Planetary temperature has likely played a significant role in these evolutionary milestones^5,6^ and will continue to influence evolution and extinction into the deep future^7^.

Conventionally, the isotopic composition of oxygen (δ^18^O) within minerals in marine fossils has served as the primary indicator for reconstructing Phanerozoic paleotemperatures^8,9^. But for the first 200 million years of the Phanerozoic (i.e., the early-to-middle Paleozoic), oxygen isotopes recorded in both carbonates and phosphates indicate a secular shift towards much lower values, implying that low-latitude seawater may have had temperatures greater than 40 °C and perhaps up to 50 °C at times during the Cambrian and early Ordovician^10–16^.

Such elevated temperatures, if accurate, would have posed lethal threats to early life forms^17,18^ and challenge our understanding of both the Paleozoic carbon cycle^19^ and background extinction rates^20^. Understandably, this interpretation of δ^18^O records has been contentious, with a long-standing debate focusing on whether the observed secular decline in isotope ratios is genuinely reflective of temperature change, or whether it instead reflects changes in the background seawater isotopic composition, potentially driven by hydrothermal activity^21–25^. A recent comparison of oxygen isotopes in different lithological classes has been applied on billion-year timescales over Earth’s history^26^, and indeed suggests a rise in seawater δ^18^O that is unrelated to temperature change. However, the degree of non-temperature-related variation over the Phanerozoic remains unclear, and even small changes in seawater oxygen isotope composition have a significant influence on temperature predictions using this proxy^27^.

In this study, we aim to shed new light on the debate around Phanerozoic temperatures by developing a globally-integrated paleotemperature reconstruction that is both completely independent of oxygen isotope ratios and is well-sampled throughout Phanerozoic time. We base our method on the Chemical Index of Alteration (CIA), which quantifies the ratio of immobile oxides such as aluminum oxide (Al_2_O_3_) to more mobile oxides (CaO, Na_2_O, K_2_O), and is a robust proxy for the intensity of weathering experienced by a geological sample^28,29^. CIA values have long been regarded as a potential proxy for regional paleo-temperature reconstruction^30–33^ and recent analyses of modern river sediments have confirmed a significant positive correlation between CIA values and local temperature^34,35^. Given that the CIA can be calculated using only major element data, which are widely gathered as part of geochemical analyses, it can be calculated for a wealth of different locations and throughout all the stages of the Phanerozoic using existing geochemical compilations.

## Results and discussion

As with other well-used temperature proxies, CIA is known to depend on other environmental factors aside from temperature. Increased physical erosion rates in a catchment decreases weathering intensity as weatherable material spends less time in the weathering zone^36^. Hydrological factors such as fluid residence time and runoff also influence CIA, particularly where groundwater reaches saturation with respect to the products of weathering reactions^37^. Nonetheless, evidence from modern river sediments suggests that climate—and particularly temperature—plays the dominant role in controlling CIA values, while other factors exert comparatively limited influence^35^. In addition, local vegetation can alter weathering characteristics by promoting the dissolution of bedrock minerals^38^. However, weathering intensity and aluminum mobility in paleosols are relatively stable across the Paleozoic evolution and expansion of vascular plants^39,40^. This stability may reflect compensating effects of plant-enhanced mineral dissolution versus increased cation retention in thicker biologically stabilized soils, together with biologically mediated weathering on pre-vegetated land surfaces by organisms such as cyanobacteria^41–43^. Overall, we acknowledge that the CIA proxy has clear uncertainties, but note that similar types of uncertainties are associated with oxygen isotope proxies. Oxygen isotope proxies for most of the Phanerozoic are derived from shallow-marine carbonates, which record regional rather than global conditions, and are influenced by non-temperature factors including salinity and, as noted above, the oxygen isotopic composition of seawater, which is very poorly constrained. Different oxygen isotope methodologies therefore give Paleozoic global average temperature predictions that diverge by around 20 °C^12,16,23^.

In our approach, we first gathered a comprehensive dataset of major element compositions from global modern river sediments from 91 peer-reviewed references (*n* = 3768; see “Methods” and SI for full references) to establish the relationship between CIA and the mean annual surface temperature at the sample location. Comparing the CIA measurements to local temperatures derived from the high spatial resolution global weather data, HydroATLAS^44^, we found a strong positive linear relationship between sample CIA and the local temperature (*r*^2^ = 0.91, *p* < 0.001, Supplementary Fig. 6), reinforcing the CIA-temperature linkage reported from other smaller datasets^34,35^.

For our paleotemperature reconstruction, we compile local observations of CIA from the geological record and convert these into local paleotemperatures at their paleolatitudes and paleolongitudes. We then use a stage-by-stage suite of coupled Atmosphere-Ocean General Circulation Model (AOGCM) HadCM3L simulations at variable CO_2_ levels^45^ to infer the modelled global mean surface temperature (GMST) that most closely reproduces the inferred local temperature at each individual location. This method, which has been previously applied to the Cretaceous-Eocene^46^, ultimately produces thousands of individual estimates of GMST across the Phanerozoic and then combines them to form an uncertainty window for likely global average temperatures over time. This procedure allows us to reconstruct GMST using scattered data gathered from very different climate zones. The underlying assumption in our approach is that the relationship between local and global temperatures in the climate model is realistic when averaged across multiple locations.

**Fig. 1:**
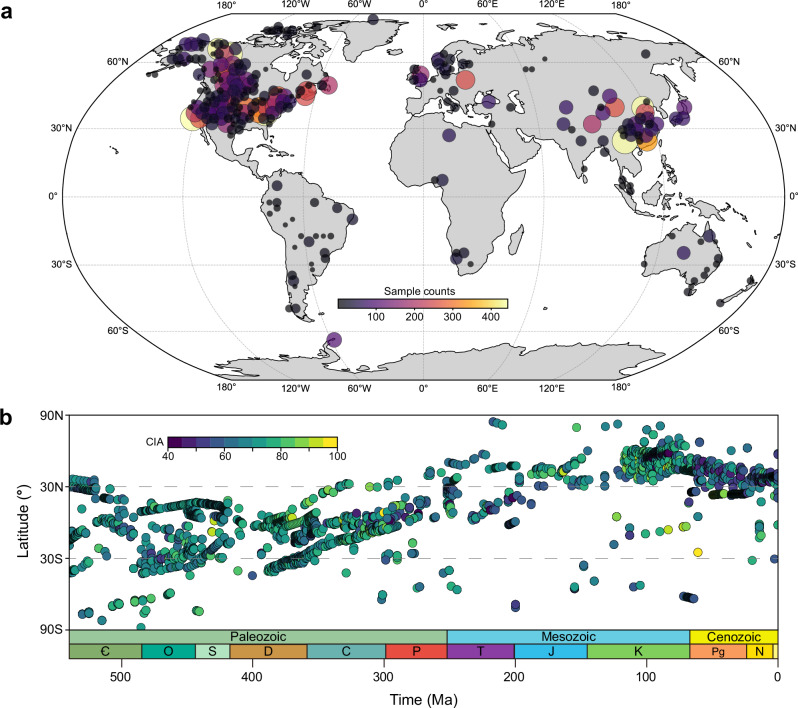
Modern and Phanerozoic spatial distributions of the Sedimentary Geochemistry and Paleoenvironments Project (SGP)^47^ and Cenozoic Chemical Index of Alteration (CIA) compilations. **a** Modern geographic distribution of CIA records from the SGP database^47,48^ and the expanded Cenozoic compilation. **b** Temporal and paleogeographic distribution of Phanerozoic CIA records. All samples are restored to their paleogeographic positions using the PALEOMAP model^52^. Є Cambrian, O Ordovician, S Silurian, D Devonian, C Carboniferous, P Permian, T Triassic, J Jurassic, K Cretaceous, Pg Paleogene, N Neogene. Ma, million years ago. The full CIA records are provided in Supplementary Data S2.

To reconstruct Phanerozoic paleotemperatures, we average the results from three regression methods between local temperature and CIA values, including the linear regression model defined from our large sediment datasets (Supplementary Fig. 6), along with the existing linear regression models from other datasets^34,35^. We then analyze a substantial collection of siliciclastic sedimentary rock records spanning the whole Phanerozoic Eon, obtained from the Sedimentary Geochemistry and Paleoenvironments Project (SGP)^47^ and its most recent Phase 2 release^48^, as well as an additional Cenozoic literature compilation (see SI). This dataset (*n* = 18,368 after data cleaning, see “Method” for details) has a wide spatial distribution across the Phanerozoic Eon (Fig. 1; see Supplementary Figs. 22–S28 for paleo-distributions). By employing the synthesized regression model and a K-metasomatism correction^49–51^ (full procedures and validations in “Methods”; Supplementary Figs. 3–S5), we translated the individual CIA values in the SGP dataset into corresponding local surface air temperatures. We utilized the PALEOMAP paleogeographic model^52^—the same as used in the AOGCM—to restore our Phanerozoic samples accurately to their paleogeographic locations. Admittedly, CIA-temperature relationships may exhibit non-linear behavior under extreme conditions, e.g., saturation at very high CIA values. However, such effects are limited in our dataset and are unlikely to affect the first-order climate trends (see “Method” for further discussion).

**Fig. 2:**
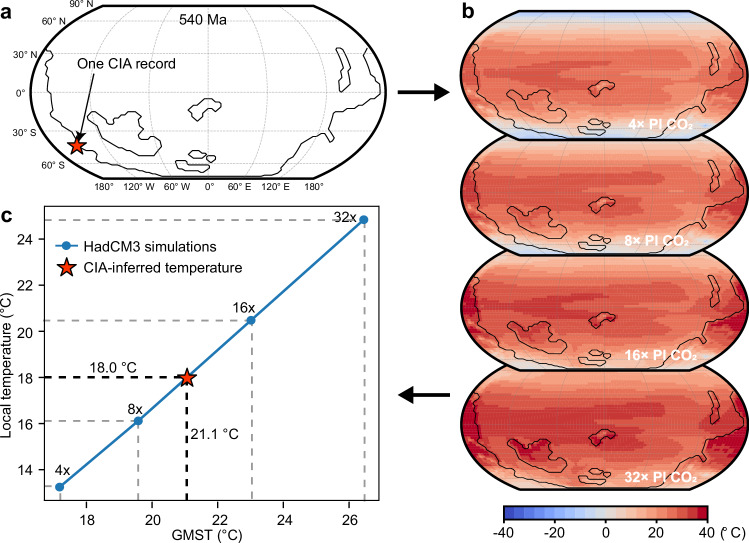
Workflow illustrating the assimilation of CIA proxy records with HadCM3L simulations to reconstruct global mean surface temperature (GMST). **a** Example of a 540 Ma CIA record restored to its paleogeographic location. **b** HadCM3L surface temperature fields simulated/extrapolated under 4–32× pre-industrial (PI) CO₂ forcing. **c** Schematic showing the mapping of CIA-derived local temperatures to GMST. Ma, million years ago.

We then compared each local paleotemperature prediction with the local predictions from the AOGCM, which has been run for 1, 2, and 4× preindustrial (PI) CO_2_ levels (1× PI CO_2_ level being defined as 280 ppm) as well as for a proxy-derived CO_2_ history^53^ (see “Methods”). These four temperature fields are further interpolated and extrapolated to encompass 10 distinct atmospheric CO_2_ concentrations ranging from 0.5 to 256 times preindustrial levels (Supplementary Figs. 10 and S11). For each sample, the local CIA-derived temperature was matched to the AOGCM simulations by linear interpolation across adjacent CO₂ scenarios, and the corresponding GMST was estimated from the same interpolated model state (Fig. 2). This results in 18,368 individual estimates for GMST over Phanerozoic time, from which we calculate percentiles. Although changes in latitudinal sampling density occur through time, particularly the dominance of Cenozoic samples from North America in the SGP database, such sampling heterogeneity is addressed by incorporating additional published dataset (Fig. 1; see “Methods”) and by our approach. Specifically, each data point is being used to select the best-fitting interpolated global climate model state at that location only, rather than being used as a direct estimate of global temperature^46^. We tested the ability of this procedure to reconstruct preindustrial GMST from limited scattered CIA datasets, as will be the case in the geological record. Using CIA records from only 36 unique geographic locations (corresponding to a 2.5° latitude × 3.75° longitude area)—the minimum number of distinct CIA locations available at the period level in our geological compilation—and with these locations either centered around North America or distributed globally, we found that our method was able to accurately reconstruct the preindustrial GMST from this limited CIA data, with mean errors of 0.52 and 1.36 °C for the global and North America cases respectively (Supplementary Fig. 7).

**Fig. 3:**
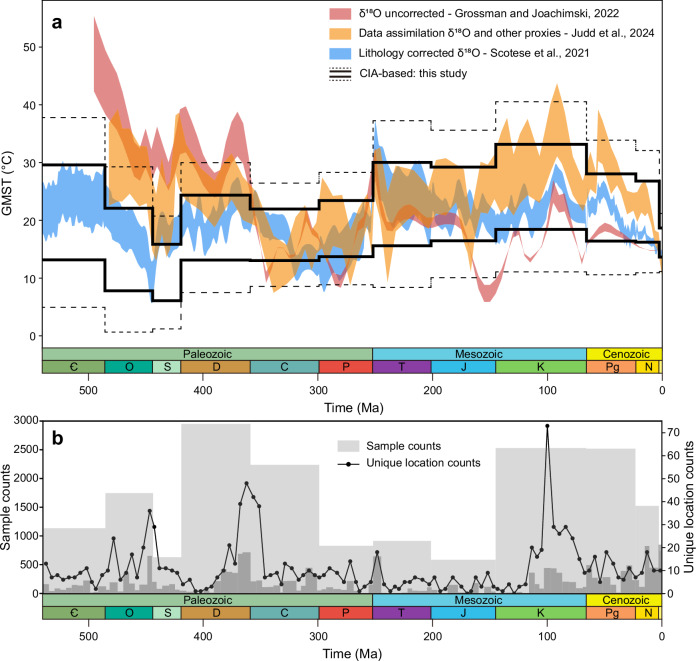
Phanerozoic GMST estimates. **a** GMST estimates from this study are shown as black lines; the solid and dashed lines represent the central 50% range and full range after excluding outliers, respectively. Outliers are defined as values beyond 1.5× the interquartile range (IQR). The upper and lower limits of this GMST estimation are determined by averaging results based on three regression methods and potassium-metasomatism correction following method in Fedo et al.^49^, Panahi et al.^50^, and Algeo et al.^51^. The regression method between CIA and local temperature averaged linear regression model defined for the modern river sediments, as well as two existing linear regression models from modern samples^34,35^. The blue band shows GMST estimations based on the lithology-corrected oxygen isotope data from Scotese et al.^54^, with the uncertainty associated with the Paleo–Köppen classification, following van de Meer^97^. The orange band represents the climate model simulation-corrected oxygen isotope records from Judd et al.^16^ (5–95 percentile). The red band represents the global sea surface temperature (SST) reconstructions and associated uncertainty based on uncorrected oxygen isotopes, converted from tropical SST estimates by Grossman and Joachiski^12^, considering a global-to-tropical SST change ratio of 1.325 ± 0.255^98^. **b** CIA sample counts and unique location counts. The light gray histogram exhibits the sample counts at Period level; the dark gray histogram and the dotted line show the sample counts and unique sample locations at 109 stage-level time intervals according to the paleogeographic configurations in Scotese and Wright^52^. A “unique” sample location is defined by a unique grid cell within HadCM3L, corresponding to a 2.5° latitude × 3.75° longitude area. The full CIA records, local temperature estimates, and associated GMST estimates are provided in Supplementary Data S2 and S3.

Figure 3b shows sample counts at the period and stage level, as well as the unique locations sampled for each stage through the Phanerozoic Eon. Due to low data abundance for some stages, we base our global interpretation on the period level, where each period contains data from ≥521 samples and ≥36 locations. By looking at the period level, we also reduce the impact of short-term hyperthermal events that have punctuated the Phanerozoic, in order to focus on the long-term trends. We plot the full spectrum of GMST estimates from this approach, which is relatively wide, and also show the central 50% of predictions. The 109 individual maps of CIA-inferred local temperatures for each geologic stage are shown in Figs. S22–S28.

Our estimation of the Phanerozoic GMST is compared to three recent oxygen isotope approaches (Fig. 3a). The method of Grossmann and Joachimski^12^ was not corrected for any potential drift in seawater δ^18^O value, while the method of Judd et al.^16^ was corrected by considering a relatively small ~1 per mill (‰) increase of δ^18^O_sw_ since the Ordovician, as well as climate model data assimilation to account for sampling location. The method of Scotese et al.^54^ was corrected for a possible large drift in seawater δ^18^O by normalizing oxygen isotope temperatures to broad lithological climatically sensitive indicators, including the locations of ice caps, coals, and evaporites. The uncorrected and minimally-corrected records predict hot (>55 °C) early Paleozoic seawater, whereas the corrected method produces much more moderate estimates, with maximum temperature of ~30 °C.

**Fig. 4:**
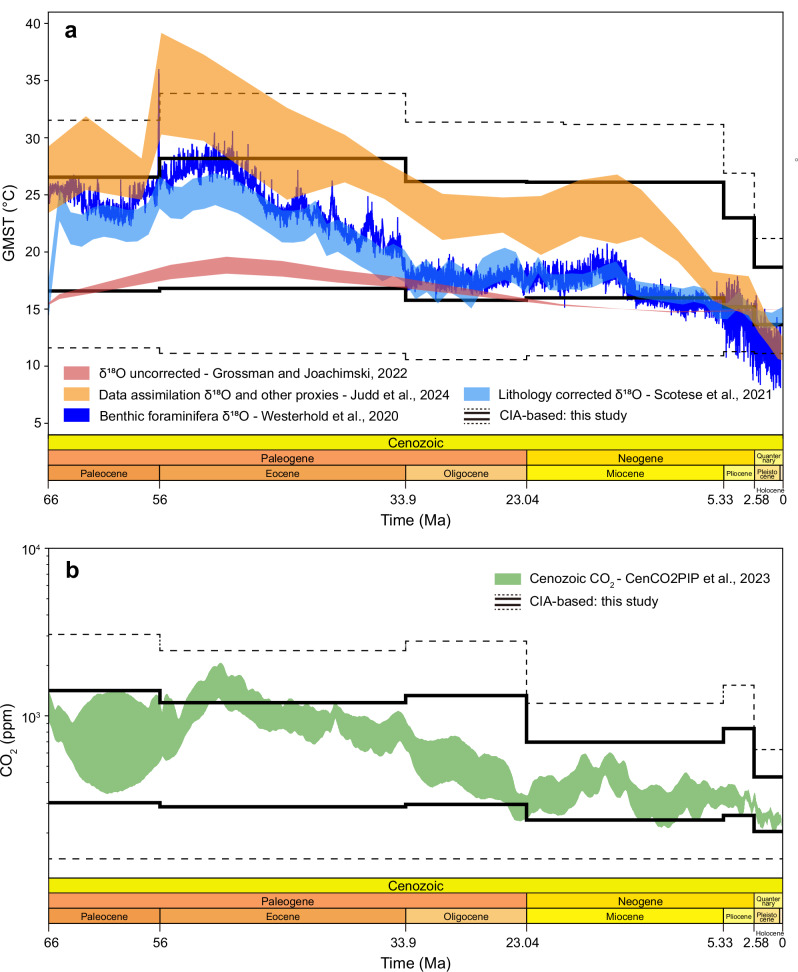
Cenozoic GMST estimates and CIA-inferred CO₂ levels at the epoch level. **a** GMST estimates from this study are shown as black lines; the solid and dashed lines represent the central 50% range and full range after excluding outliers, respectively. The blue band, dark blue line, orange band, and red band represent Cenozoic GMST estimates from Scotese et al.^54^, Westerhold et al.^8^, Judd et al.^16^, and Grossman and Joachiski^12^, respectively. **b** CIA-inferred Cenozoic CO_2_ levels. The inferred CO_2_ levels from this study in black lines; the solid and dashed lines represent the central 50% range and the full range after excluding outliers, respectively. The green band represents the proxy-based CO_2_ reconstructions (2.5th–97.5th percentile) in CenCO2PIP et al.^55^. The full CIA records, local temperature estimates, and associated GMST estimates are provided in Supplementary Data S2 and S3.

Our estimate is broader in time than published oxygen isotope methods, and includes more uncertainty, given the broad timescale binning and the other non-temperature factors that can influence CIA values. Nevertheless, our GMST reconstruction clearly picks out the expected cooling into the Ordovician, Carboniferous, and Cenozoic icehouses. To further evaluate the fidelity of our reconstruction, we test the CIA-based approach in more detail for the Cenozoic, where independent temperature^8^ and CO₂ constraints^55^ are relatively well established and data is more abundant. The expanded Cenozoic compilation integrates the SGP dataset with CIA data from 36 peer-reviewed papers, yielding a total of 4788 Cenozoic CIA records (Fig. 1 and Supplementary Data S2). Using this enlarged dataset, we reconstructed Cenozoic GMST at the epoch level. The resulting GMST captures well-established Cenozoic temperature trends, including early Paleogene warmth followed by long-term cooling, as demonstrated by the close agreement between the central 50% CIA-derived GMST estimates and independent GMST reconstructions (Fig. 4a). In addition, CIA-inferred atmospheric CO₂ levels are consistent with independent proxy-based reconstructions^55^ (Fig. 4b). These results indicate that, despite relatively large uncertainty bounds, this CIA-based framework is capable of recovering robust global climate signals when sufficient data is available.

Even under the more conservative conditions imposed by the low temporal resolution in deep time, our Phanerozoic-scale results lead to a clear conclusion that the “corrected” oxygen isotope method of Scotese et al.^54^ shows a far better agreement with our approach than the “uncorrected” methods. Particularly, we find that the uncorrected oxygen isotope curve sits outside our maximum error bounds during most of the Cambrian-to-Devonian interval. This means that not a single one of the thousands of CIA samples for these times supported a local surface temperature compatible with the uncorrected oxygen isotope curve. The minimally-corrected oxygen isotope curve of Judd et al.^16^ (which uses oxygen isotopes for the Paleozoic and combines these with other records for the late Mesozoic to Cenozoic) is a closer fit, but still falls almost entirely above the central 50% of our estimation. The corrected δ^18^O curve of Scotese^54^ falls within our central 50% estimate for almost all of the Cambrian to Devonian. During the late Paleozoic and for the Meso- and Cenozoic, the CIA method predicts temperatures that more closely overlap with the previous methods, which themselves also converge more clearly. This is encouraging because the effect of drift in seawater oxygen isotope values should diminish towards the present day, which should cause the various corrected and uncorrected δ^18^O methods to converge, and to track non-δ^18^O records.

In conclusion, our findings indicate that the Cambrian and early Ordovician were likely not warmer than other warm periods during the Mesozoic or Cenozoic eras. Lithological maps support this, showing a restriction of bauxite deposits^56^, and warm-water carbonate formation and reefs at low latitudes^57^ rather than extension closer to the poles that might be expected if tropical temperatures were 40–50 °C. A hypothesized “hot Cambrian” is followed by the glaciated late Ordovician, and such a dramatic reduction in global temperature has been shown to be incompatible with our currently-understood carbon cycle models^19^. Furthermore, Sea Surface Temperatures >35 °C are fatal for the phytoplankton^58,59^ that underpin the modern marine food chain and temperatures > 40 °C are fatal for multicellular eukaryotes, seemingly contrasting with the continuous occurrence of animal fossils (including in the tropics) throughout the early Paleozoic^60,61^. Further, these high temperatures are even less likely to support metazoan life when considered in conjunction with the reduced early Paleozoic atmospheric oxygen levels. Aerobic habitability for ectothermic animals such as inhabited the early Paleozoic can be limited by low oxygen levels (O_2_ supply) or by high temperatures (which increase metabolic rates, e.g., O_2_ demand)^62^. Both Earth system modeling^63^ and geochemical data^64^ suggest the Cambrian and Ordovician were characterized by lower-than-modern levels of atmospheric oxygen, which may have varied between ~10 and 60% of present atmospheric levels. Our more moderate temperature record for the early Paleozoic can be reconciled with these lower atmospheric oxygen levels and the continual existence of animals, whereas local temperatures of 40–50 °C cannot. Notably, however, some of the biologically important temperature trends seen in δ^18^O records also appear in our CIA-based records (albeit at coarser resolution), specifically the dramatic cooling over the Cambrian-Ordovician. This supports hypotheses linking global cooling to the Ordovician radiation^14,15^, specially under lower levels of atmospheric oxygen, where decreases in temperature would dramatically increase aerobic habitability.

Overall, our results support the idea that the Earth is able to strongly regulate surface temperature through a network of negative feedback processes—the most important of these likely being climate-dependent silicate weathering^65–67^. This regulation contributed to maintaining global average surface temperatures between approximately 10–30 °C during the Phanerozoic, supporting conditions for life to flourish. Other studies have observed that weathering intensity in sediments and paleosols has stayed within a broadly similar range over 3 + Ga^39,40,68^, suggesting that our inference of stable climate could be extended further back in time^26^. Given the apparently tight regulation of temperature over the whole Phanerozoic, and considering the sporadic evidence for glaciation throughout Earth’s history, the global average surface temperature of Earth over geologic timescales may have never varied far from the values recorded in the Phanerozoic, a situation which likely contributed to the continued diversification of life.

## Methods

### Calculation of the chemical index of alteration and data acquisition from the SGP dataset

The CIA is a widely used proxy for rock weathering. It evaluates weathering intensity by comparing the ratio of labile cations (Ca²⁺, Na⁺, K⁺) to the immobile cation Al³⁺. High CIA values indicate extensive weathering through the removal of labile cations, while lower values suggest limited weathering with less removal of these cations^28^. The CIA value is calculated as follow:

1 $${{\rm{CIA}}}=\frac{{{{\rm{Al}}}}_{2}{{{\rm{O}}}}_{3}}{{{{\rm{Al}}}}_{2}{{{\rm{O}}}}_{3}+{{\rm{Ca}}}{{{\rm{O}}}}^{*}+{{{\rm{Na}}}}_{2}{{\rm{O}}}+{{{\rm{K}}}}_{2}{{\rm{O}}}}\times 100$$

where all oxides are measured in molar units rather than weight percent, with CaO* representing the portion of CaO within the siliciclastic fraction.

We collected Phanerozoic CIA records from the Sedimentary Geochemistry and Paleoenvironments Project (SGP)^47^ and its most recent Phase 2 release^48^. The SGP is a community sedimentary geochemical database spanning Earth’s history^47,48^, comprising 126,006 samples with 4,132,371 reported analytical results. For this study, the complete Phase 2 dataset was downloaded from the SGP Phase 2 database (https://sgp-search.io/; data API - “show”: [“alu”, “ca”, “k”, “na”, “min_age”, “max_age”, “interpreted_age”, “interpreted_age_notes”, “coord_lat”, “coord_long”, “section_name”, “strat_name”, “lithology_name”, “data_source”, “analysis_ref_short”; last accessed on 05/Dec/2025). After data filtering and cleaning (see procedures below), the SGP dataset contains 15,664 CIA records. We expanded the Cenozoic coverage by compiling an additional 2704 CIA records from clay- and silt-sized siliciclastic rocks reported in 36 peer-reviewed studies (see SI). The integrated Phanerozoic dataset comprises a total of 18,368 CIA records. We then restored the Phanerozoic samples to their paleogeographic locations using PALEOMAP paleogeographic reconstructions, consistent with those employed in the HadCM3L climate simulations, implemented via the GPlately scripts^69^. This data compilation provides broad spatial coverage across the Phanerozoic Eon (Figs. 1 and S22–S28).

### Data cleaning and K-metasomatism correction

1. Initial filtering and lithological screening. We first filtered to include only Phanerozoic samples, with interpreted ages ranging from 540 to 0 Ma. We excluded samples missing major element composition, interpreted ages, or coordinates. To minimize the effects of grain size and hydraulic sorting, we retained only fine-grained siliciclastic lithologies, including argillite, claystone, clay, meta-argillite, metapelite, mud, mudstone, silt, siltstone, metasiltstone, pelite, shale, slate, oil shale, and siltite. Consequently, only fine-grained siliciclastic records were retained for analysis. Because depositional environment information (e.g., terrestrial or marine) is not systematically reported in the SGP records, the dataset may include samples from both settings. However, given their shared detrital weathering origin, such variability is unlikely to introduce systematic bias into the derived CIA-temperature relationship^68,70,71^. After obtaining the dataset, the element compositions are converted into wt% oxides before the CIA calculation, which used the molar units of the oxides.

2. Carbonate and recycling screening. Because carbonate addition can artificially lower CIA values and produce unrealistically low temperature estimates^68,72^, we removed samples with CaO contents greater than 8 wt%, following methods in Lipp et al.^68^. Moreover, CaO* is used to exclusively quantify silicate-related CaO, thereby minimizing bias. We adopted McLennan’s method^73^ for this purpose, equating CaO (silicates) to Na_2_O if the molar quantity of CaO, post-apatite correction, exceeds that of Na_2_O. To ensure CIA values reflect contemporaneous primary silicate weathering, we excluded samples explicitly described in their original publications as recycled or derived from pre-weathered sedimentary sources (e.g., Dumoulin and White^74^; Young^75^; Campbell et al.^76^). We further exclude extreme outliers beyond 1.5× the interquartile range (IQR; i.e., below Q1 − 1.5×IQR or above Q3 + 1.5×IQR) to minimize the influence of sporadic anomalous samples that were likely affected by sedimentary recycling or other post-depositional overprints.

3. K-metasomatism correction. K-metasomatism refers to the post-depositional alteration process in which potassium (K), often introduced as K⁺ ions from pore fluids, is added to sedimentary rocks, leading to an artificially elevated K₂O content^49^. This alteration can significantly bias geochemical weathering proxies such as the CIA by increasing the denominator (i.e., K₂O), thereby lowering CIA values and potentially underestimating the degree of original chemical weathering—and consequently, the reconstructed temperatures in this study. The LOWESS regression of the (CaO + Na₂O)/K₂O (CN/K) molar ratio across all samples and A-CN-K ternary diagrams suggest samples, especially before Cenozoic, experienced some degree of diagenetic K enrichment (Supplementary Figs. 1 and S2).

We performed a correction following the method of Fedo et al.^49^ and using the formulation of Panahi et al.^50^, which adjusts K₂O to recover pre-metasomatic compositions. This method requires knowledge of the molar fraction of K₂O in the unaltered parent-material. Because protolith compositions are unknown for most samples in our global deep-time compilation, we adopt the average Upper Continental Crust (UCC) composition as a proxy^77^ (Al₂O₃ = 15.4 wt%, Na₂O = 3.28 wt%, CaO = 3.60 wt%, K₂O = 2.81 wt%), yielding a parent-material molar K fraction of ~0.10 (i.e., the parameter *m* in Eq. (1) of Panahi et al.^50^). After applying the Panahi correction, we compared measured and corrected K₂O values and retained the smaller of the two, as the corrected value represents the inferred pre-metasomatic composition and should not exceed the measured value if *K* addition occurred. Cases where the correction yields higher K₂O values are interpreted as artefacts of applying a generalized UCC protolith to locally variable source rocks and are therefore not considered physically meaningful. This treatment is consistent with Algeo et al.^51^, who emphasize that K-metasomatism can follow multiple alteration vectors, and that the pure-addition endmember assumed by the Fedo–Panahi correction likely overestimates *K* enrichment in many natural systems where partial replacement of CaO + Na₂O dominates.

As a result, the K-corrected CIA values should be interpreted as upper-bound estimates, whereas uncorrected CIA values represent the lower-bound case in which diagenetic effects are assumed to be minimal (Supplementary Fig. 3). The impact of K-metasomatism correction is particularly pronounced for early Paleozoic records, with GMST estimates for the Ordovician, Silurian, and Devonian shifting upward and showing improved agreement with glacial records (Fig. 3a). This interpretation is supported by the relatively low values of (CaO + Na₂O)/K₂O observed during these intervals (Supplementary Fig. 1), indicating a greater susceptibility to *K* addition. We therefore use the corrected CIA values for our primary GMST reconstruction (Fig. 3a), while explicitly acknowledging that the true values likely lie between the corrected and uncorrected endmembers.

We further evaluate whether variations in UCC could influence the GMST reconstruction. Although the average composition of the UCC is generally considered to have remained relatively stable through the Phanerozoic^78^, estimates vary among different compilations and rock types. Because the potassium correction applied to CIA values depends on the parent-material molar *K* fraction, such variations could potentially influence the reconstructed temperatures. To assess this effect, we conducted a sensitivity analysis using several representative UCC compositions. The average UCC composition reported by La Maitre et al.^79^ (Al₂O₃ = 14.6 wt%, Na₂O = 3.98 wt%, CaO = 4.32 wt%, K₂O = 2.95 wt%) yields a parent-material molar K fraction of 0.10, consistent with Rudnick et al.^77^. In contrast, the Paleozoic UCC composition proposed by Gaschnig et al.^80^ (Al₂O₃ = 11.2 wt%, CaO = 4.8 wt%, Na₂O = 1.6 wt%, K₂O = 2.4 wt%) produces a higher K fraction of 0.14, whereas a weathered young UCC composition^81^ (Al₂O₃ = 18.24 wt%, Na₂O = 0.79 wt%, CaO = 1.12 wt%, K₂O = 1.17 wt%) yields a lower value of 0.08 due to its higher Al₂O₃ content and reduced Na₂O, CaO, and K₂O. These estimates define a plausible range of parent-material *K* fractions (0.08–0.14). Recalculating CIA values and GMST using these end-member scenarios results in only minor changes in absolute GMST values, while the overall Phanerozoic warming and cooling trends remain unchanged (Supplementary Figs. 4 and S5). This suggests that reasonable variations in parent-rock composition exert only a limited influence on the reconstructed first-order GMST evolution.

### Data acquisition of the large global CIA dataset and correlation between CIA and Temperature

The relationship between CIA and temperature has been established based on average CIA values and temperatures from forty-four modern estuary sediments^34^ (Temperature = 0.56 × CIA–25.7, *R* = 0.70) and a global compilation of modern sediment dataset from 46 peer-reviewed literatures^35^ (Temperature = 0.98 × CIA–58.07, *R* = 0.99).

To further expand the dataset, we collected additional major element oxide data from modern river sediments (*n* = 3768) documented in peer-reviewed papers, supplementing the datasets of Deng et al.^35^ and Müller et al.^82^ (*n* = 91; see Supplementary Data S1). Consistent with prior research^34,35^, and to control for grains effects^83^, our analysis focused exclusively on riverine silty and clay sediments (from channels or riverbanks). We calculated CIA values for these samples using the methodology outlined in the previous data cleaning section. To examine the relationship between CIA values and temperature, we use annual mean surface air temperature data from WorldClim v1.4^84^, extracted at the same locations as the CIA records. These data, as documented in the HydroATLAS dataset^44^, are provided at ~1 km spatial resolution (30 arc-s). Linear regression analysis suggested a strong positive correlation between CIA values and temperatures (Supplementary Fig. 6), with an *R*^2^-value of 0.91 and *p*-values < 0.0001. The linear regression equation is defined as:

2 $${{\rm{Temperature}}}=0.93\times {{\rm{CIA}}}-52.51$$

This finding aligns with previous studies that correlated CIA values of modern river sediments with modern temperatures^34,35^. We note that the updated WorldClim v2 dataset^85^ provides similar spatial patterns and global mean annual temperature values to WorldClim v1.4. Test regressions using WorldClim v2 produced results nearly identical to those obtained with WorldClim v1.4. Therefore, we retained WorldClim v1.4 in this study to maintain methodological consistency with previous results^34^.

We estimated best-guess global temperatures for all Phanerozoic CIA samples in the SGP dataset by comparing CIA-derived local temperature estimates with corresponding local temperatures from interpolated HadCM3L simulations. We then translate CIA-derived temperatures into GMST values from the same interpolated HadCM3L climate state. Admittedly, the linear regression models used in this study cannot fully capture potential non-linear relationships between chemical weathering intensity and temperature. For example, samples with very high CIA values may become insensitive to further temperature increases once the proxy approaches saturation. However, only 206 samples (~1% of the total dataset) in our dataset have CIA values above 90, and none occur in the early Paleozoic interval. Therefore, although such non-linear effects may exist, they are unlikely to significantly affect the first-order GMST trends reconstructed in this study.

### Climate model simulations

The reconstruction method is underpinned by an ensemble of model simulations at multiple CO_2_ concentrations, at 109 discrete intervals through the Phanerozoic. We use a version of the coupled AOGCM, HadCM3^86^. The specific underlying version, HadCM3BL-M2.1aD, is described in detail in Valdes et al.^87^, with additional modifications applied in order to simulate deep-time past climates, described in Valdes et al.^45^. The model has a horizontal resolution of 3.75 × 2.5° in longitude–latitude in both the atmosphere and the ocean, and a vertical resolution with 19 unequally spaced vertical levels in the atmosphere and 20 in the ocean. The model generates paleo-temperature data for each individual paleogeographic map, running for a minimum of 5000 model years to approach close to a dynamic equilibrium. In addition to the paleogeographic configurations, HadCM3L also incorporate solar constant and atmospheric CO_2_ concentrations as time-dependent boundary conditions. The solar constant is based on Gough^88^ and increases linearly at an approximate rate of 11.1 W m^−2^ per 100 Myr to 1365 W m^−2^ currently. For each of the 109 time periods, 4 sets of simulations are carried out. The first set is identical to the “Foster” simulations of Valdes et al.^45^, for which atmospheric CO_2_ is prescribed according to the values in Foster et al.^53^. The other three sets are at constant CO_2_ values of 1× preindustrial values (i.e., 280 ppm), 2× (560 ppm), and 4× (1120 ppm). These simulations were all used in the GMST reconstruction work of Judd et al.^16^, where they are named scotese_02, scotese_solara, scotese_2co2a, and scotese_4co2a, respectively (https://zenodo.org/records/8237751).

### Validation on the CIA-model assimilation using modern CIA records

To assess the robustness of our GMST estimation method, which assimilates CIA-inferred temperatures with AOGCM simulation outputs, we perform a validation using modern CIA records and present-day HadCM3L simulations. Considering that the minimum number of distinct CIA locations available at the period level in our geological compilation is 36, we randomly select only 36 global CIA records (Supplementary Fig. 7a), along with their corresponding temperatures, to identify the best-matching HadCM3L scenario among CO₂ levels ranging from 0.5 to 256× PI CO₂. Repeating this procedure 1000 times yields a mean GMST error of 0.52 ± 0.95 °C (Supplementary Fig. 7b) relative to the pre-industrial GMST of 15 °C.

We further validate the method by limiting the CIA records to North America. Since the majority of CIA records from the Cretaceous to the present in SGP dataset are derived from North America. We randomly select 36 CIA records within 10° to 85° latitude and −170° to −50° longitude (Supplementary Fig. 7c), then repeat the CIA-model assimilation procedure 1000 times. The resulting GMST error is 1.36 ± 0.84 °C (Supplementary Fig. 7d), differing by less than 1 °C from the GMST estimation using global CIA records. Moreover, the GMST estimates based solely on North American data largely encompass the pre-industrial reference value of 15 °C, suggesting that our data assimilation procedure is robust and only minimally influenced by geographic sampling bias.

### Temperature reconstructions from the average regression model and CIA-inferred CO_2_ levels

Although the three linear regression methods—each defined using datasets of different sizes—yield different equations, the resulting temperature reconstructions are largely consistent. The temperature reconstruction using linear method based on the large compilation of modern river sediments produces the warmest upper temperature limits, in which the early Paleozoic and Cretaceous global temperature can reach up to 31 °C and 34 °C within the central 50% estimate, with an absolute maximum of ~40 °C. Even when choosing this warmest reconstruction method and observing the upper extreme of the error window, it is not possible to recreate the extreme Cambrian temperatures suggested in oxygen isotope estimations (Supplementary Fig. 8). For the best-guess estimate in the manuscript, we average all of these regression methods, including linear regression model in this study and other two published linear models^34,35^ (Fig. 3a).

We also compare the CO₂ ranges predicted by our CIA–simulation assimilation framework for each period with independent CO₂ reconstructions derived from proxy records^53,55^ and biogeochemical models^89,90^. We find that the central 50% CO₂ estimates obtained in this study closely match both proxy- and model-based reconstructions, and the upper confidence bounds show good agreement with proxy and model records during the early Paleozoic (Supplementary Fig. 9).

### Assessing uncertainty in GMST estimation and validation

1. Uncertainty related to model extrapolation. In our paleotemperature reconstruction, CIA samples were first restored to their original latitudes and longitudes. We then derived their predicted local temperatures using the defined linear regression model, followed by comparing these local proxy temperatures to HadCM3L simulations conducted at varying CO_2_ levels. This approach enabled us to estimate the GMST that best correlated with each local proxy, introducing an uncertainty range for GMST over time (Fig. 3a). This uncertainty primarily arises from the diversity of CIA samples across different periods.

HadCM3L^87^ is a coupled atmosphere-ocean global climate model. It uses the 109 paleogeographic configurations of the PALEOMAP project^52^ but at a lower resolution (3.75° × 2.5° in longitude × latitude). In this study, we have employed four different scenarios of atmospheric CO_2_ concentrations, including 1, 2, 4× pre-industrial (PI) CO_2_ levels, and CO_2_ volumes described in Foster et al.^53^. To allow for very high possible surface temperatures, we extend the HadCM3L simulations by extrapolating them via 2D piecewise linear interpolation to produce temperatures under 10 different CO_2_ scenarios, ranging from 0.5 to 256× PI CO_2_ levels (Supplementary Fig. 10).

Extrapolating model estimates to such high surface temperatures increases uncertainty. We note that some of these extrapolations result in unrealistic inverted temperature gradient at high latitudes, e.g., large temperature inversions in the high latitudes or steep high latitudinal temperature gradients. This inverted temperature gradient occurs when there is no contiguous landmass situated over a pole, due to the effects of continentality, the difference in the heat capacity between water and landmass, and the freezing point of seawater. Under such conditions, high latitudes may exhibit temperatures comparable to, or even slightly higher than, those at lower latitudes. (e.g., 2× and 4× PI scenario in 315 Ma; Supplementary Fig. 11). This inversion could be further amplified under higher CO_2_ conditions but only affects less than 2% (~400 records) of the total CIA records, which estimate GMST based on these inverted temperatures. Similar inversion has been documented in recent oxygen isotope-model assimilation studies^16^. This issue, induced by extrapolation, is adjusted especially for the early Paleozoic, because the HadCM3L simulation is conducted at higher than 4× PI CO_2_ levels—reaching peaks over 2000 ppm (equivalent to 7.14× PI CO_2_)—until 391 Ma, as atmospheric CO_2_ volumes described in Foster et al.^53^. Therefore, extrapolation of surface temperatures at any periods later than 391 Ma can only be performed with the incorporation of HadCM3L simulations at 1, 2, and 4× PI CO_2_ levels. Accordingly, the early Paleozoic period exhibits a smoother latitudinal temperature gradient, with fewer instances of inversion (Supplementary Fig. 11). In contrast, the latitudinal temperature gradient of Mesozoic or Cenozoic Era still exhibits unrealistic temperature gradients, shown by the abnormal temperature patterns at high latitudes.

Paleoclimate models for the Phanerozoic are not typically run at extreme temperatures, but runs of another coupled ocean and atmosphere GCM, the Fast Ocean Atmosphere Model (FOAM) climate model for 128× CO_2_ in the Cambrian show a Cenozoic-like temperature gradient, with warmer equator and cooler poles^91^ (Supplementary Fig. 12). We therefore (1) employed a scale factor (Supplementary Fig. 13) and (2) flatten the inverted temperature gradient to address the inverted latitudinal temperature gradient. We introduced a parabola scale factor. This factor adjusts for the differential spatial responses to increasing CO_2_ levels over time (illustrated in Supplementary Fig. 13). The scale factor is defined as follows:

3 $${{\rm{Scalefactor}}}=1-\left[{0.25\times \left(\frac{{{\rm{latitude}}}}{90}\right)}^{2}\right]$$

This scale is set to 1.0 at the equator and gradually decreases to 0.75 at the poles, reflecting a decreasing trend from equator to poles. This adjustment is designed to further reduce instances of inverted temperature gradients, although it does not eliminate them entirely (Supplementary Fig. 14). Nevertheless, combined with the previous adjustment, this method further cools the average temperatures across the Phanerozoic Eon, showing that low temperatures in our reconstruction do not depend on over-extrapolation of the climate model results (Supplementary Fig. 15).

We also adjusted the inverted temperature gradient by flattening the temperature gradient in the extrapolated scenarios when mean zonal temperature from middle-high latitudes (>|50°|N/S) is warmer than mean zonal temperature from lower latitudes. This procedure can exclude the inverted temperature gradient (Supplementary Fig. 16). Compared to the GMST without this adjustment, the GMST estimates only slightly changed (Supplementary Fig. 17). After both adjustments, the updated GMST estimations (Supplementary Figs. 15 and S17) are very similar to the previous GMST estimation in Fig. 3a. These tests suggest that our climate model extrapolation does not alter our conclusions—because the recorded CIA values do not support such extreme temperatures.

To further validate the 2D interpolation of HadCM3L simulated temperatures, we analyzed the latitudinal temperature gradients from FOAM simulations. The FOAM model was simulated at CO_2_ levels as high as 128× PI for the Cambrian Period^91^, providing a suitable comparison for our HadCM3L interpolations. The latitudinal temperature gradient from high CO_2_ FOAM simulations reveals a consistent increasing pattern from the poles to the equator (Supplementary Fig. 12), paralleling our interpolated HadCM3L results. The highest average temperature observed in the FOAM simulations at 128× PI CO_2_ is 37 °C, with a minimum of 21 °C and a maximum of 46 °C (Supplementary Fig. 18). Notably, the average, minimum, and maximum temperatures demonstrate an approximately linear increase of 4 °C for each increment in CO_2_ levels (Supplementary Fig. 18). Based on this pattern, we hypothesize that a GMST of 50 °C requires CO_2_ level of 1024× preindustrial. Furthermore, the minimum pole temperature in the 128× PI CO_2_ FOAM simulation exceeds 21 °C, aligning with the pole temperature estimations from the HadCM3L simulations. Assuming the observed linear temperature increment with rising CO_2_ levels, polar temperatures could exceed 33 °C when the GMST reaches 50 °C. Given that CO_2_ infrared absorption bands become progressively saturated at high concentrations^92^, such an extreme CO_2_ level would yield diminishing radiative forcing and is physically unrealistic. This reinforces our interpretation that even during the warmest intervals of Earth’s history, global mean temperatures were likely constrained below extreme values.

2. Uncertainty related to grain size. Grain-size variations may influence CIA values, as coarse-grained (e.g., sandy) sediments typically exhibit lower CIA values than fine-grained, clay-rich sediments due to dilution by quartz and feldspar^93,94^. In this study, we primarily selected silty- to clay-sized siliciclastic rocks to reduce potential grain-size effects. To further evaluate this influence, we used the Al₂O₃/SiO₂ ratio as a proxy for grain size^95^, where higher ratios generally indicate clay-rich sediments and lower ratios reflect quartz-rich, coarser sediments. The temporal distribution of Al₂O₃/SiO₂ across the Phanerozoic shows no systematic long-term trend (Supplementary Fig. 19a), suggesting that the dataset is not strongly biased toward particular grain-size fractions through time. In addition, the correlation between CIA and Al₂O₃/SiO₂ is relatively weak (*r* = 0.33; Supplementary Fig. 19b), indicating that grain-size sorting is unlikely to be the primary control on CIA values.

To further assess this effect, we applied a conservative filter by excluding samples with Al₂O₃/SiO₂ <0.1, which likely represent quartz-rich, coarse-grained sediments. The GMST reconstruction based on this filtered dataset (Supplementary Fig. 20) shows nearly identical warming and cooling patterns to those presented in Fig. 3a. This comparison suggests that grain-size effects do not exert a strong influence on the reconstructed first-order GMST trends, although some uncertainty may remain.

3. Uncertainty related to reverse weathering. Authigenic clay formation in marine environments, such as reverse weathering, may influence CIA values by incorporating cations (e.g., K, Na, and Ca) into newly formed clay minerals, thereby modifying the apparent degree of chemical weathering recorded by sediments. However, directly quantifying the contribution of reverse weathering is challenging, as mineralogical and geochemical constraints required to identify such processes are not consistently available in the compiled dataset. In addition, depositional environments (marine versus terrestrial) are not uniformly reported, further limiting a direct assessment of this effect.

To partially evaluate its potential influence, we conducted a sensitivity analysis based on depositional setting. Specifically, we reconstructed GMST using a subset of samples interpreted as terrestrial from paleogeographic reconstructions. In this approach, a sample is classified as terrestrial only if the surrounding 3 × 3 grid cells (approximately 11.25° longitude × 7.5° latitude) are all identified as land in the Scotese paleogeographic maps^52^, providing a conservative filtering criterion that reduces the inclusion of nearshore or potentially marine-influenced environments. The GMST reconstruction based on this terrestrial-only subset (Supplementary Fig. 21) shows broadly similar long-term trends compared to that derived from the full dataset, although some differences are observed (e.g., slightly lower temperatures during the Carboniferous, more consistent with glacial conditions). These results suggest that while reverse weathering and related marine processes cannot be fully excluded, their influence is unlikely to dominate the first-order temperature trends reconstructed in this study.

We acknowledge that CIA-based GMST reconstructions carry substantial uncertainties, arising both from limited spatial coverage in deep time and from local non-climatic controls on CIA values. Reducing these uncertainties is an important direction for future work. One effective strategy is to further expand the CIA database in both spatial and temporal coverage. As demonstrated by our Cenozoic validation (Fig. 4), applying this approach to intervals with dense, well-distributed CIA records allows robust recovery of well-known climatic features, suggesting that increased data density in other periods could substantially reduce uncertainty and improve temporal resolution in CIA-based temperature reconstructions.

In addition, integrating complementary paleoclimate proxies offers a promising avenue to further constrain GMST estimates. Combining CIA with lithological indicators, biological proxies, and isotopic records would allow cross-validation among independent climate signals and help narrow uncertainty ranges. More advanced statistical frameworks, such as multi-proxy data assimilation or Bayesian approaches, could also explicitly reduce uncertainties and optimally weight different proxies according to their strengths and limitations.

### 109 individual maps of CIA-inferred local temperatures through the Phanerozoic

We present the full 109 maps of CIA-inferred local temperatures at stage level through the Phanerozoic (Supplementary Figs. 22–S28). The stage-level time intervals are based on the paleogeographic configurations from Scotese and Wright^52^. These maps, together with Fig. 3b, suggest that our CIA dataset from SGP is characterized by a relatively consistent number of samples per period, although the Silurian, Triassic, and Jurassic periods are less sampled. Over 95% of these maps include at least five unique sampling locations (at least 36 locations at Period level), indicating a broad geographic coverage of sampling. Notably, four-time intervals lack samples, as evident in maps, e.g., 191 Ma. Nonetheless, the dataset includes a substantial number of samples and unique sampling locations when considering at period level, ensuring robustness in our GMST estimations and their associated uncertainties over these timeframes. The early Paleozoic, particularly the Cambrian and Ordovician periods, is well represented both in terms of total sample numbers and geographic distribution, providing a relatively solid foundation for accurate GMST estimation for these periods.

When focusing on warmer climatic periods, the early Paleozoic maps (Supplementary Fig. 28) show similarities to those of the Cretaceous (Supplementary Fig. 23) and Paleogene Periods (Supplementary Fig. 22). Maps from these warmer periods consistently feature CIA-inferred temperatures exceeding 20 °C at middle and/or high latitudes. Based on the latitudinal temperature gradient, these observations suggest a GMST in the range of approximately 30–35 °C. In contrast, oxygen isotope proxies, predominantly sampled in tropical regions, have indicated local temperatures exceeding 50 °C. However, our CIA proxy data do not support such high temperatures, as we observed no CIA-inferred temperatures above 30 °C. When GMST reaches 50 °C, the equator could exceed 57 °C and poles exceed 33 °C as indicated by the FOAM simulations^91^. But none of all our CIA proxy support these temperature conditions (Supplementary Fig. 28). Consequently, our analysis suggests that the early Paleozoic was a warm period, but its temperatures were comparable to other warm periods in the Mesozoic and Cenozoic Eras.

## Supplementary information

Supplementary Information

Description of Additional Supplementary Files

Supplementary Data 1

Supplementary Data 2

Supplementary Data 3

Supplementary Code 1

Transparent Peer Review file

## Data Availability

The datasets generated and analysed in this study are available in the Supplementary Information and Zenodo repository (10.5281/zenodo.19466041)^96^. The major element composition of Phanerozoic siliciclastic rocks can also be downloaded from the Sedimentary Geochemistry and Paleoenvironments Project (SGP) at https://sgp-search.io/api. The paleoclimatic maps from the HadCM3L are available from the Bristol Research Initiative for the Dynamic Global Environment website at https://www.paleo.bristol.ac.uk/resources/simulations. Paleogeographic reconstructions were performed using GPlately (https://github.com/GPlates/gplately) according to PALEOMAP (available from https://repo.gplates.org/webdav/pmm/paleomap).

## Code availability

The code and dataset used for reproducing the results in this study are available in the Supplementary Information and Zenodo repository (10.5281/zenodo.19466041)^96^.
