# Supplementary material for: Tight regulation of Earth’s long-term temperature over Phanerozoic time
Source: Nat Commun. 2026 May 4;17:5995. doi: 10.1038/s41467-026-72672-6 (PMC13347045; doi:10.1038/s41467-026-72672-6)
Supplement: Supplementary file 1 — Supplementary Information [file 41467_2026_72672_MOESM1_ESM.pdf]

Supplementary Information for

Tight regulation of Earth's long-term temperature over Phanerozoic time

Dongyu Zheng[1,2,*], Alex G. Lipp[3], Alexander Farnsworth[4,5], Shufeng Li[6], Andrew S. Merdith[2,7], Khushboo Gurung[2], Mingcai Hou[1,*], Anqing Chen[1], Zixi Hou[1], Daniel J. Lunt[4],

Erik A. Sperling[8], Paul J. Valdes[4], Benjamin J. W. Mills[2,*]

[1] State Key Laboratory of Oil and Gas Reservoir Geology and Exploitation & Institute of Sedimentary Geology, Chengdu University of Technology, Chengdu, 610059, China

[2] School of Earth and Environment, University of Leeds, Leeds, LS2 9JT, UK

[3] Department of Earth Sciences, University College London, 5 Gower Place, London WC1E 6BS, UK

[4] School of Geographical Sciences and Cabot Institute for the Environment, University of Bristol, Bristol, BS8 1SS, UK
[5] State Key Laboratory of Tibetan Plateau Earth System, Environment and Resources (TPESER), Institute of Tibetan Plateau Research, Chinese Academy of Sciences, Beijing, 100101, China

[6] CAS Key Laboratory of Tropical Forest Ecology, Xishuangbanna Tropical Botanical Garden, Chinese Academy of Sciences, Mengla, 666303, China

[7] School of Physics, Chemistry and Earth Sciences, University of Adelaide, Adelaide, 5005, Australia

[8] Department of Earth and Planetary Sciences, Stanford University, Stanford, CA 94305, USA

*Corresponding authors:

zhengdongyu@cdut.edu.cn; houmc@cdut.edu.cn; b.mills@leeds.ac.uk

Contents:

Supplementary Figures S1-S28

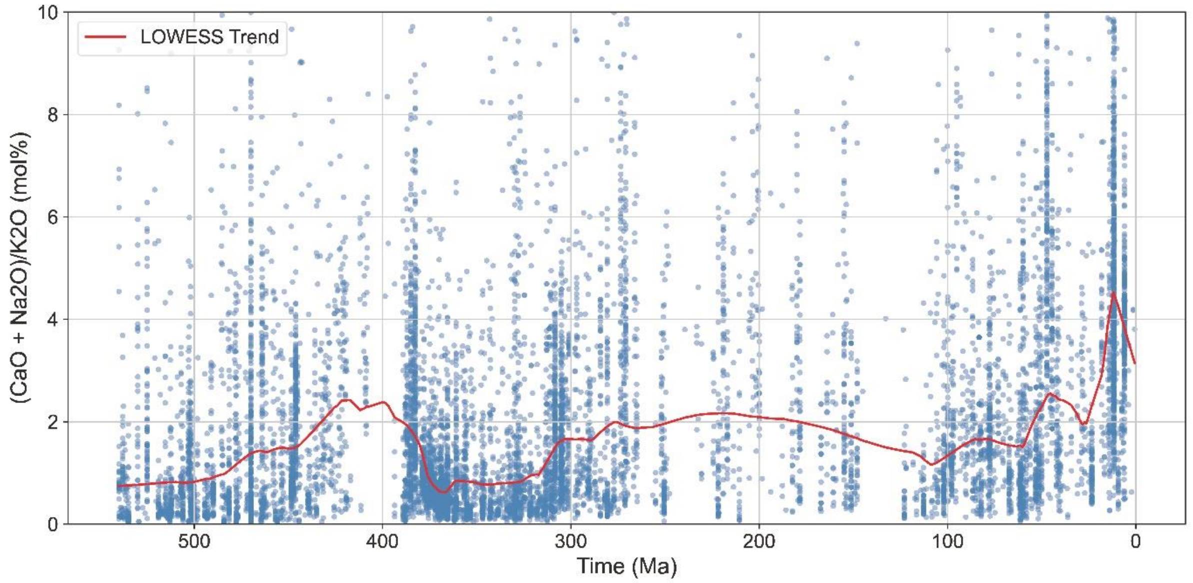

Figure S1. Molar ratio of $(CaO+Na_2O)/K_2O$ through Phanerozoic time. The red line represents the Locally Weighted Scatterplot Smoothing (LOWESS) regression curve (span = 0.1). Ma, million years ago.

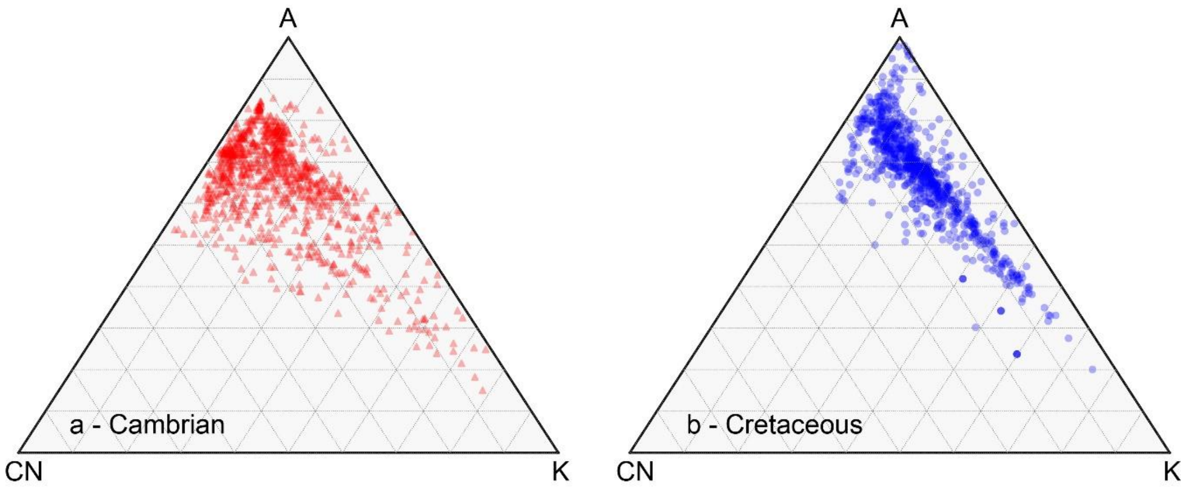

Figure S2. The A-CN-K ternary diagrams of (a) Cambrian and (b) Cretaceous samples. A–CN–K represents Al_2O_3–(CaO^* + Na_2O)–K_2O in molar proportions, where CaO* denotes CaO in silicate minerals only.

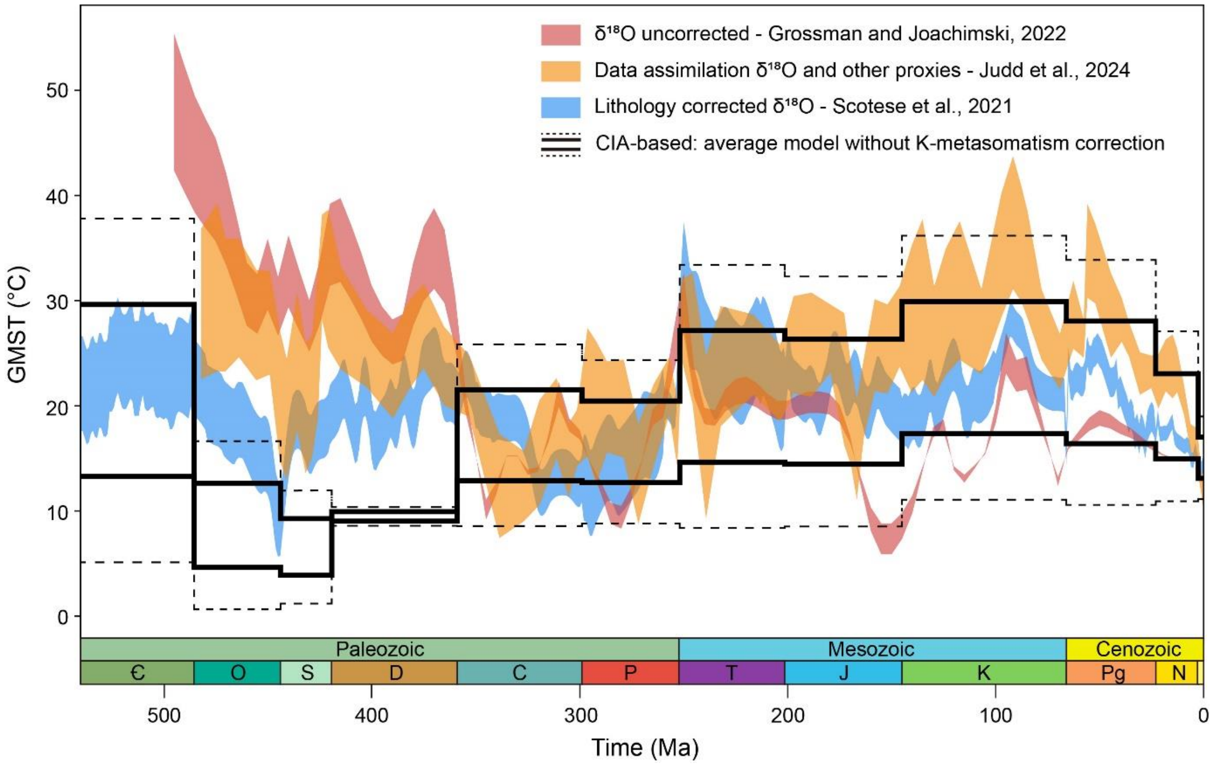

Figure S3. Global Mean Surface Temperature (GMST) estimates without potassium (K)-metasomatism correction. GMST estimations from this study in black lines; the solid and dashed lines represent the central 50% range and the full range after excluding outliers, respectively. Outliers are defined as values beyond 1.5× the interquartile range (IQR). The upper and lower limits of this GMST estimation are determined by averaging results based on three regression methods, including linear regression model defined in this study, as well as two existing linear regression models from modern samples[34,35]. The blue band, orange band, and red band represent GMST estimates in Scotese et al.[45], Judd et al.[16], and Grossman and Joachimski[12], respectively. Є: Cambrian; O, Ordovician; S, Silurian; D, Devonian; C, Carboniferous; P, Permian; T, Triassic; J, Jurassic; K, Cretaceous; Pg, Paleogene; N, Neogene.

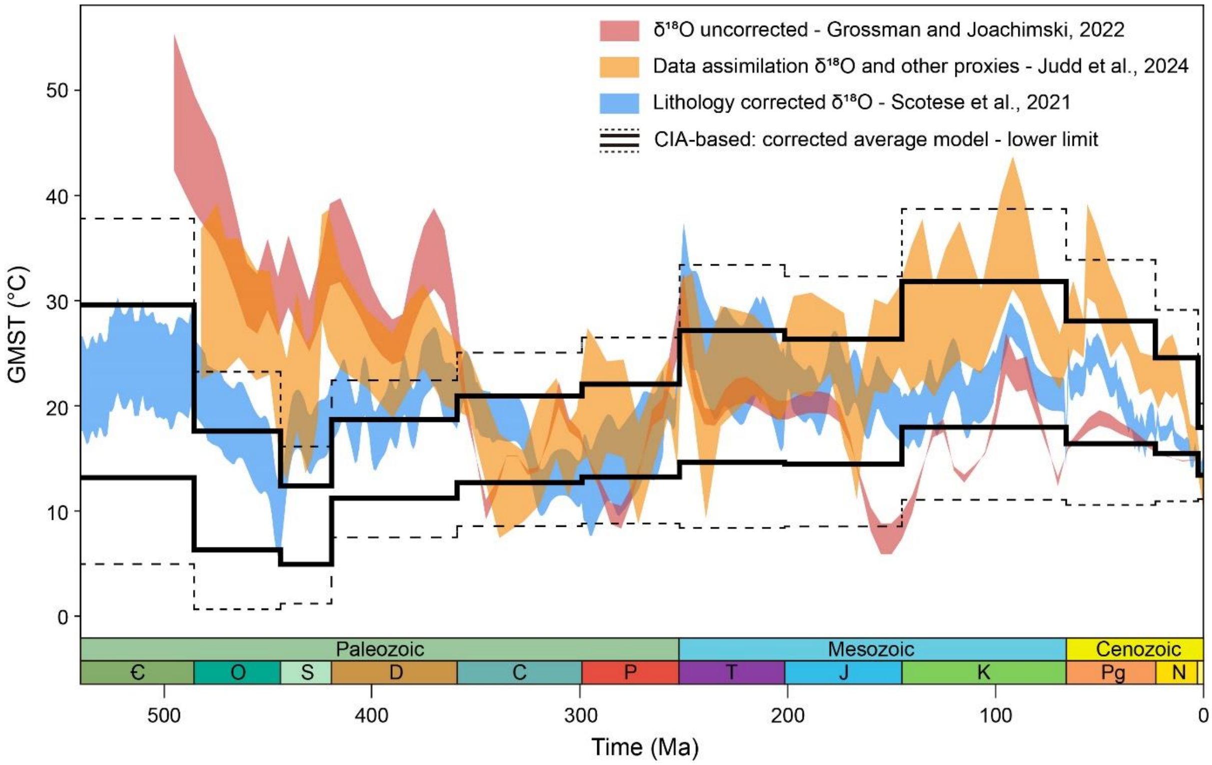

Figure S4. GMST estimates with K-metasomatism correction using a parent-material K fraction of 0.14 for the UCC. Legend as in Fig. S3.

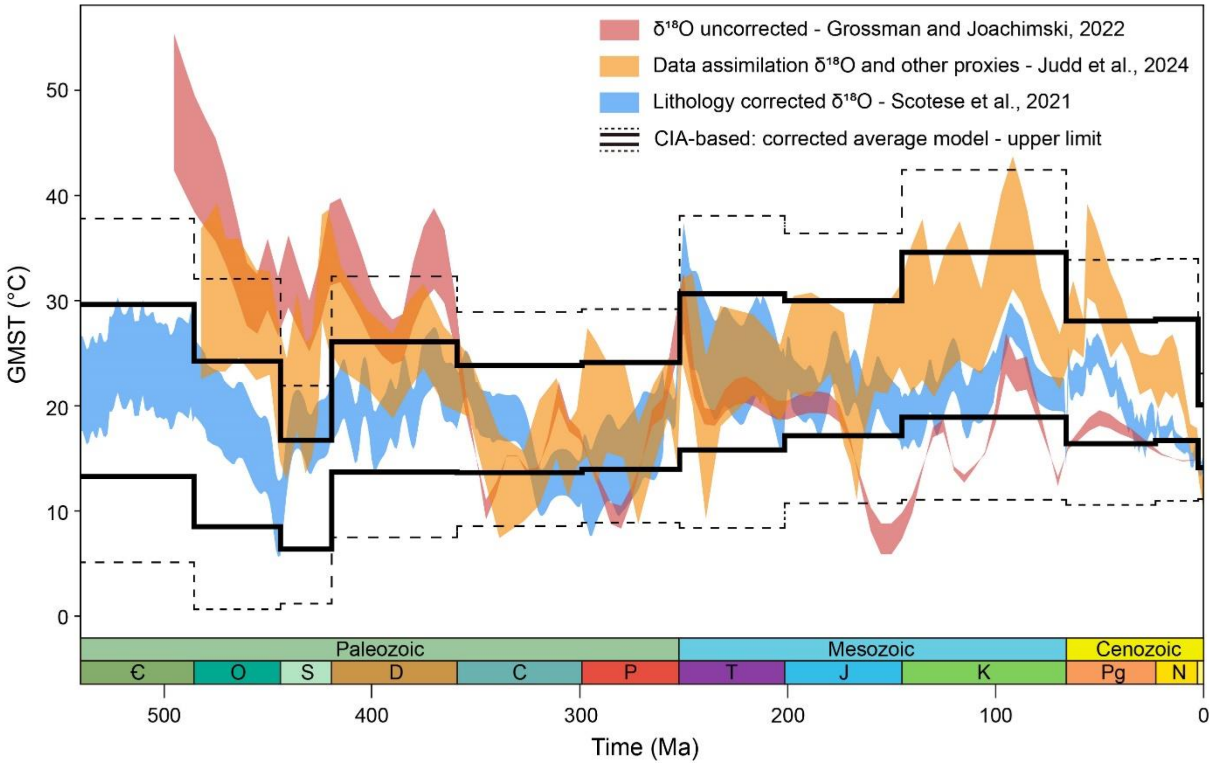

Figure S5. GMST estimates with K-metasomatism correction using a parent-material K fraction of 0.08 for the UCC. Legend as in Fig. S3.

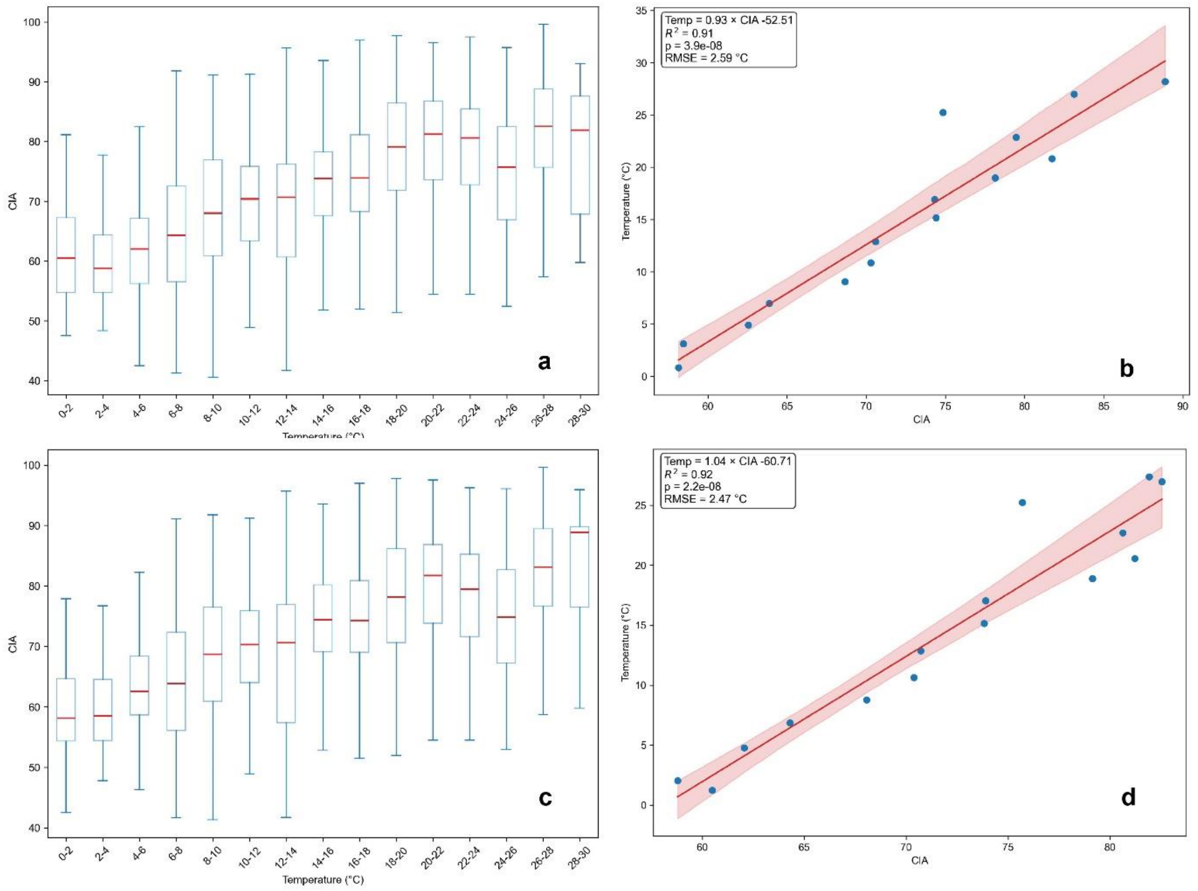

Figure S6. Correlation between global modern river Chemical Index of Alteration (CIA) and temperature. (a) Boxplots of CIA values grouped by annual mean surface air temperature in WorldClim v1.4 and (c) WorldClim v2, using 2 °C temperature bins. The boxes represent the 25th and 75th percentiles, the red line indicates the median, and the whiskers extend to the minimum and maximum values within 1.5× the interquartile range (IQR). (b) Linear regressions between CIA and temperature in WorldClim v1.4 and (d) in WorldClim v2. The regression is performed using the median CIA values of each temperature bin. The red shaded band represents the 95% confidence interval derived from 1000 bootstrap iterations. Temp, temperature; R^2, coefficient of determination; p, p-value indicating statistical significance; RMSE, root mean square error.

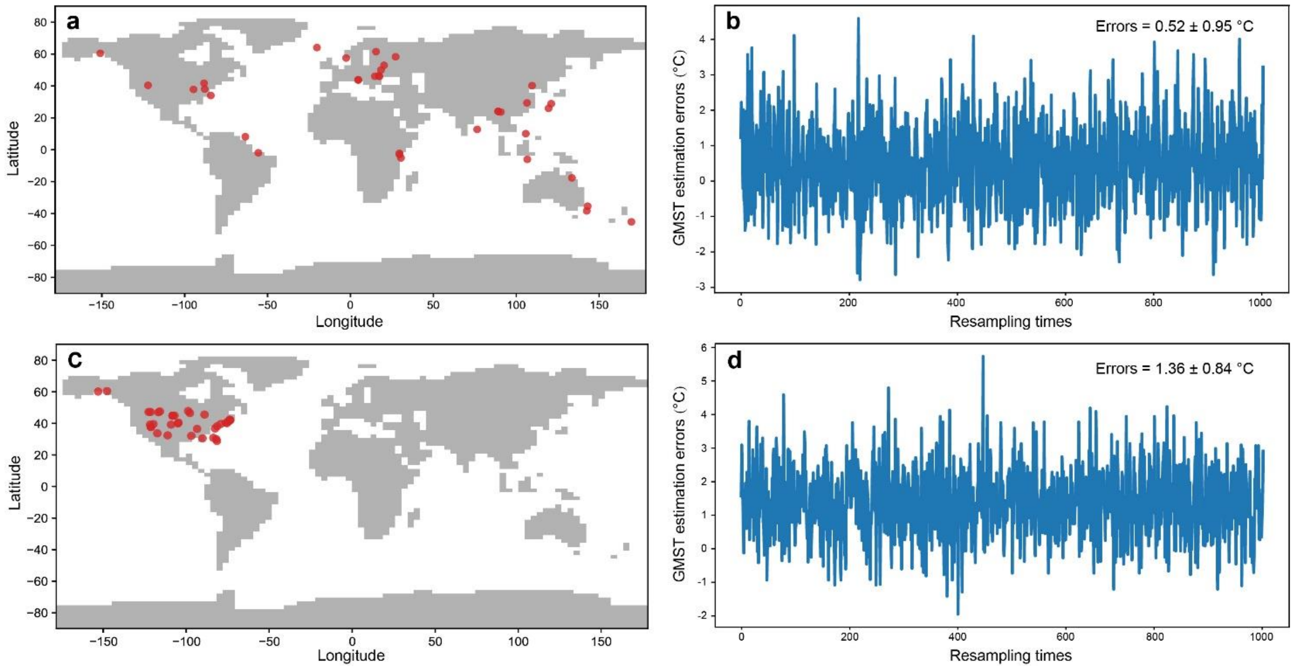

Figure S7. Modern GMST estimates using either global CIA records or North America-only CIA records. (a) GMST estimates based on 36 randomly selected global CIA records. (b) Mean and one standard deviation of GMST estimation errors from 1,000 iterations using 36 global CIA records. (c) GMST estimation based on 36 randomly selected North America-only CIA records. (d) Mean and one standard deviation of GMST estimation errors from 1,000 iterations using 36 North America-only CIA records. GMST estimation errors are calculated as the difference between the estimated GMST and the pre-industrial value of 15 °C (i.e., Error = GMST − 15 °C).

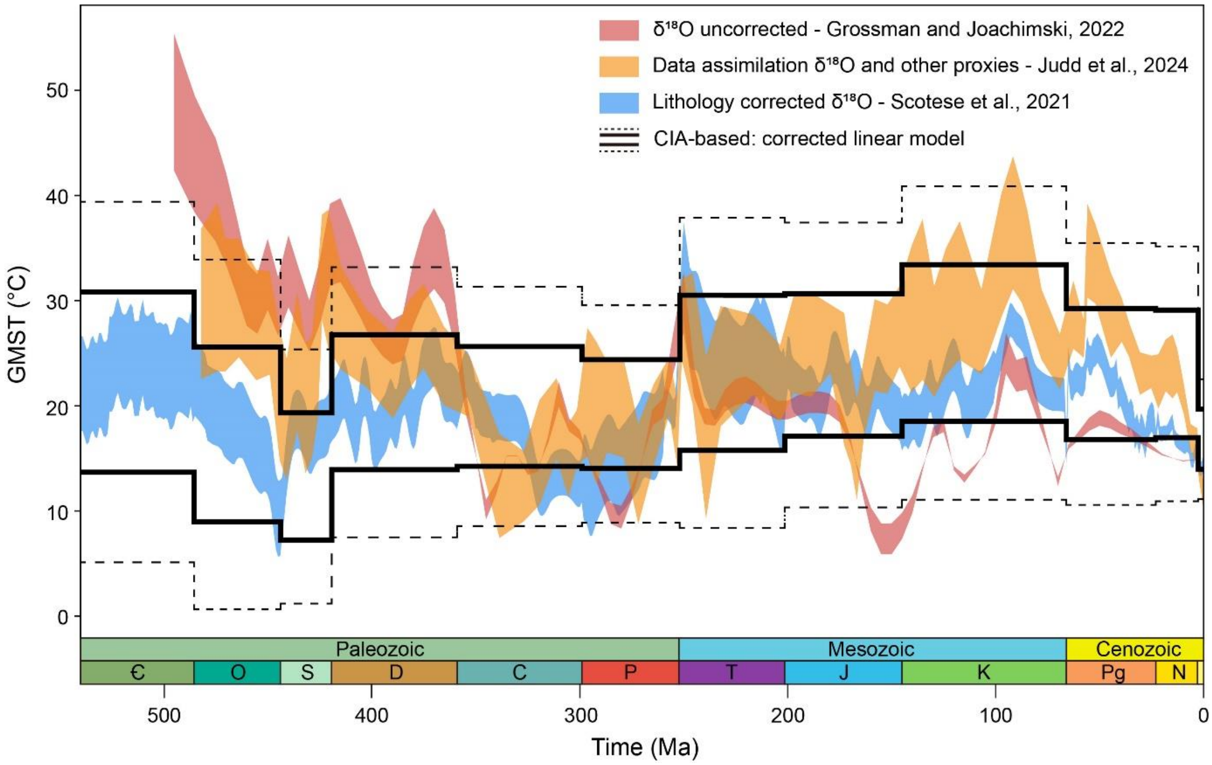

Figure S8. GMST estimates using the K-metasomatism corrected linear regression model. The linear regression model is defined in Equation 2. Legend as in Fig. S3.

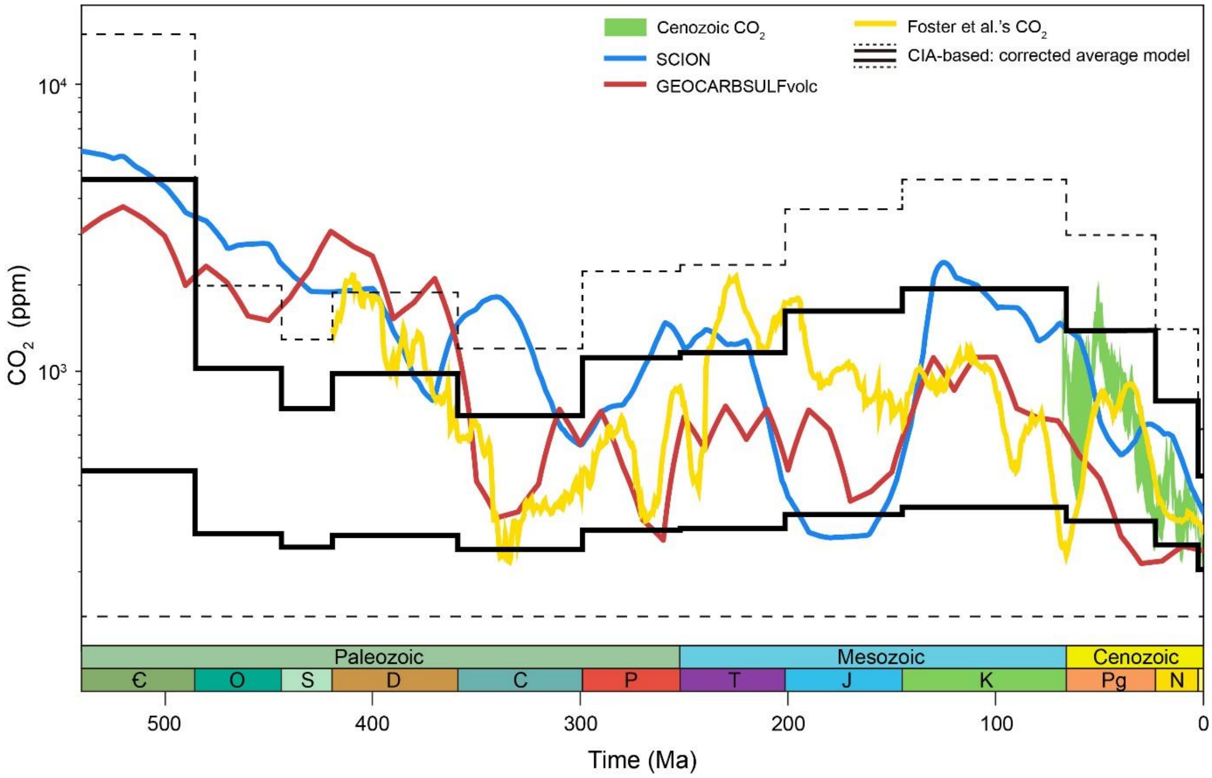

Figure S9. CO_2 ranges inferred from the CIA–simulation assimilation framework. CIA-inferred CO_2 level from this study in black lines; the solid and dashed black lines represent the central 50% range and the full range after excluding outliers, respectively. Outliers are defined as values beyond 1.5× the IQR. The green band represents the Cenozoic CO_2 estimates in CenCO2PIP et al.[55]. The blue and red lines represent model-based CO_2 estimates in Mills et al.[92] and Berner[91], and the yellow line represents proxy-based CO_2 estimates in Foster et al.[53]. Legend as in Fig. S3.

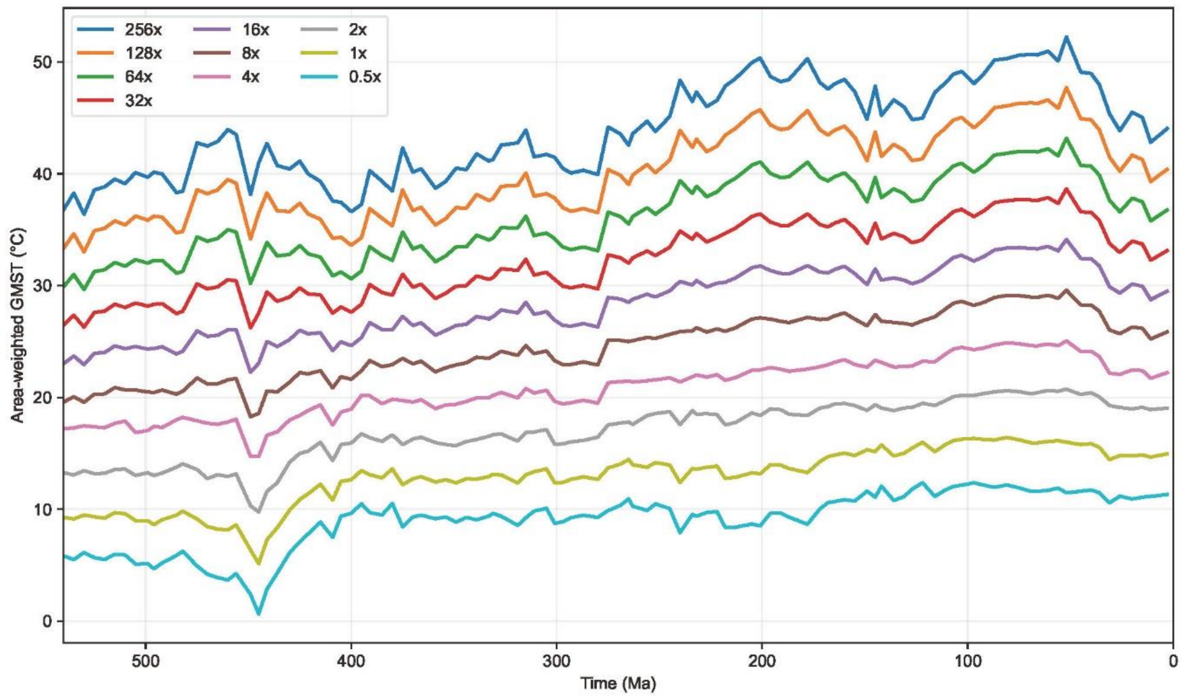

Figure S10. GMST from 2D interpolated HadCM3L simulations, integrating model runs at 1×, 2×, and 4× CO_2 and CO_2 estimates from Foster et al.[53]. Values from 0.5× to 256× represent multiples of pre-industrial (PI; 280 ppm) CO_2.

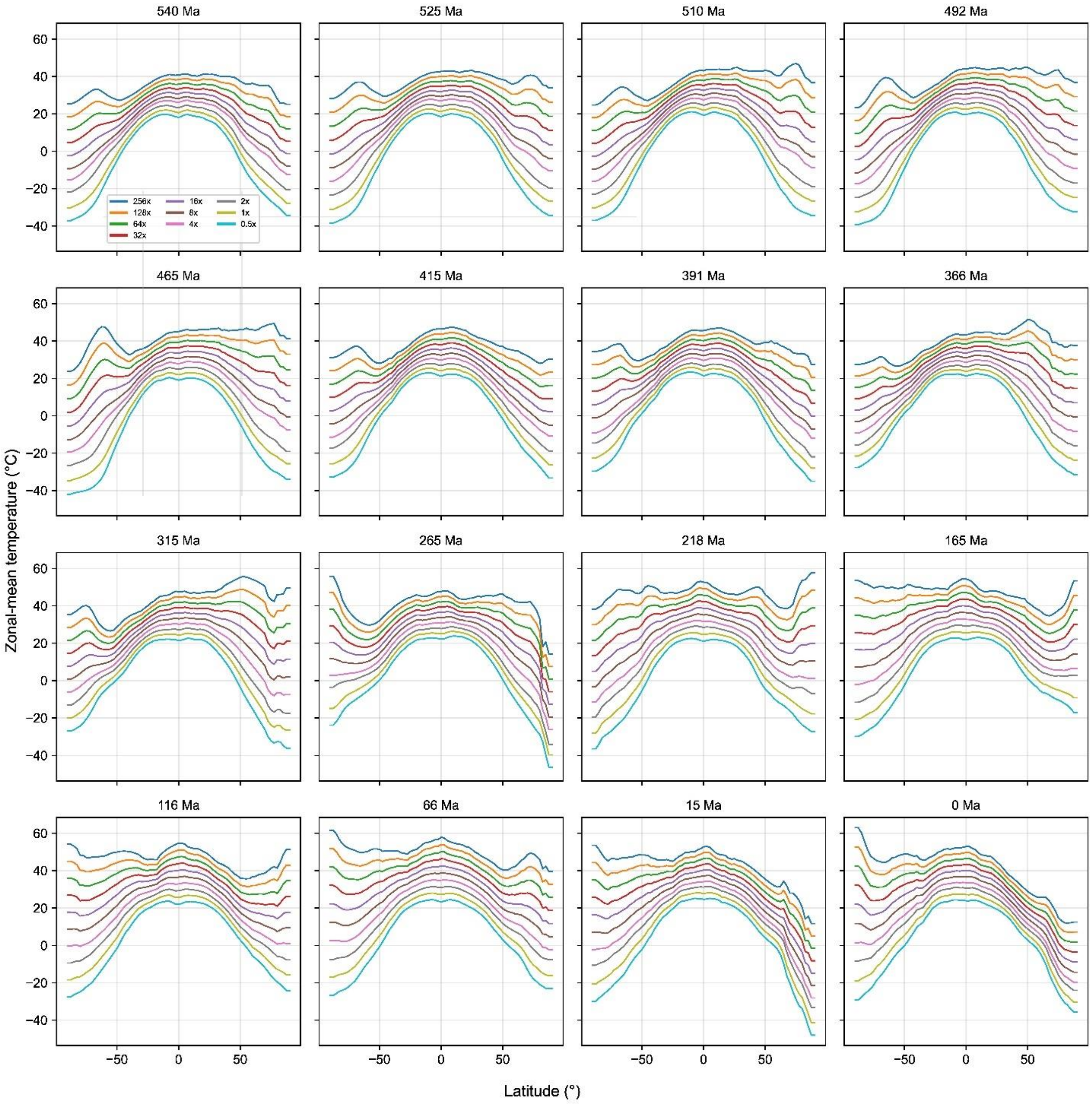

Figure S11. Latitudinal temperature gradients from 2D interpolated HadCM3L simulations under 0.5× to 256× PI CO_2 scenarios.

.

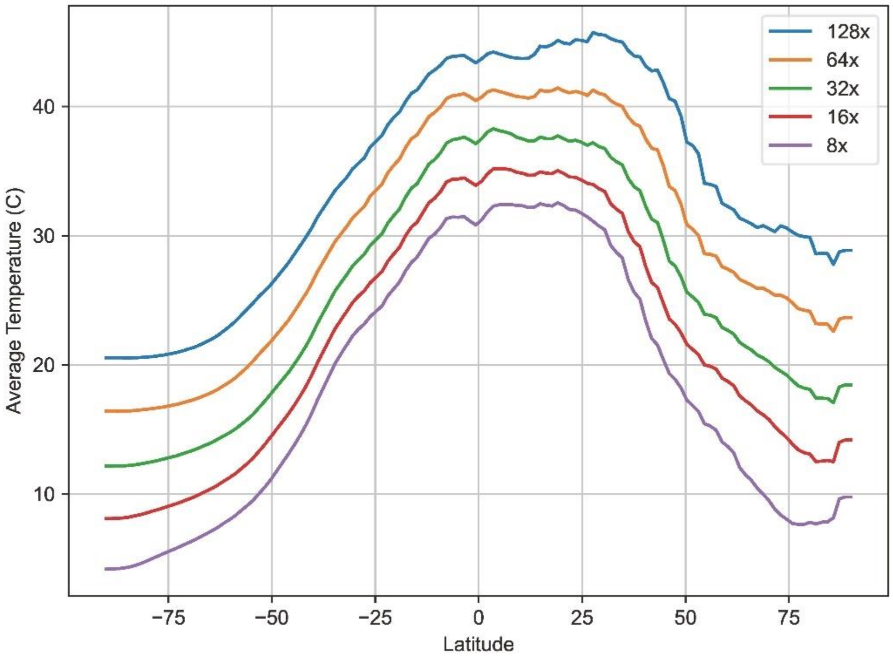

Figure S12. Latitudinal temperature gradients from FOAM simulations under 8× to 128× PI CO_2 scenarios during the early Cambrian.

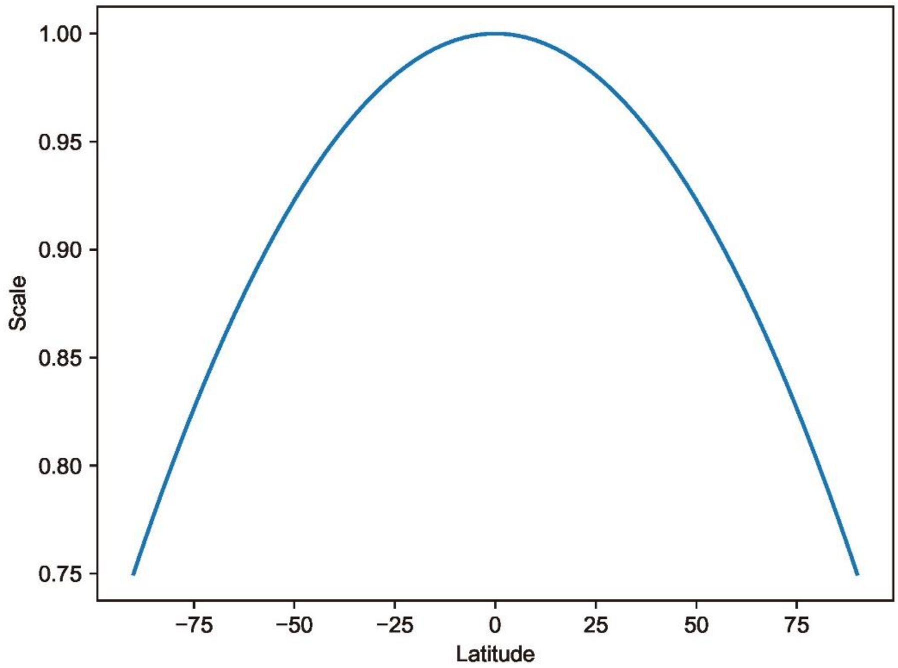

Figure S13. The scale factor used to adjust interpolated HadCM3L temperatures.

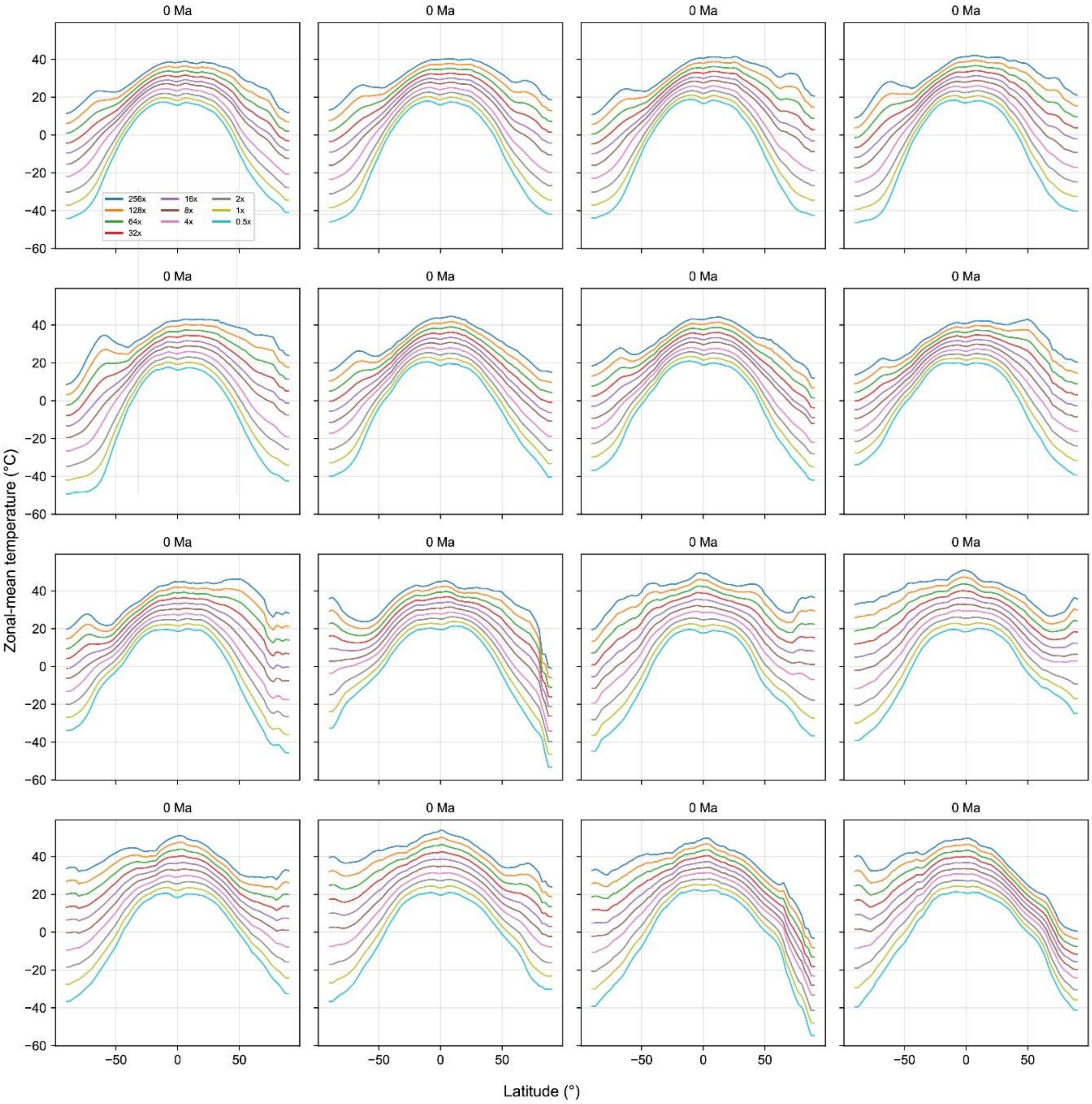

Figure S14. Latitudinal temperature gradients from 2D interpolated HadCM3L simulations with scale factor applied, across 0.5×–256× PI CO_2 scenarios.

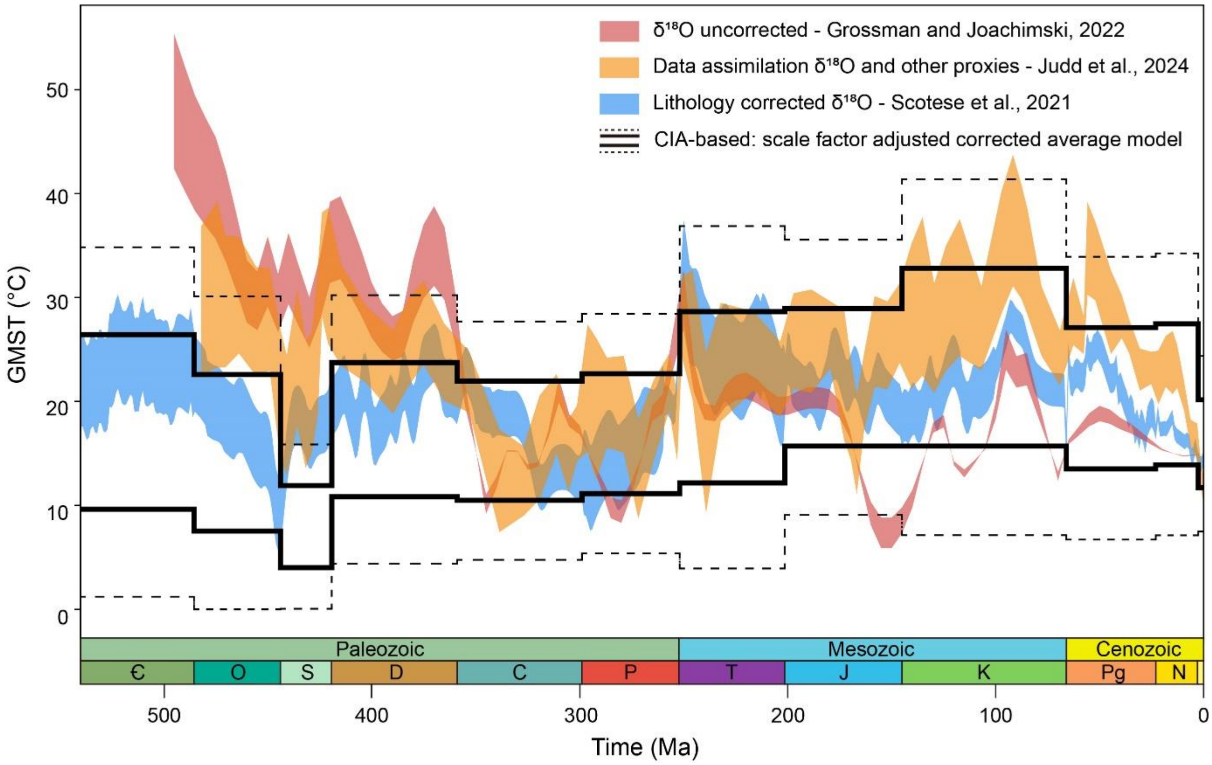

Figure S15. GMST estimates based on the scale factor adjusted HadCM3L simulations. Legend as in Fig. S3.

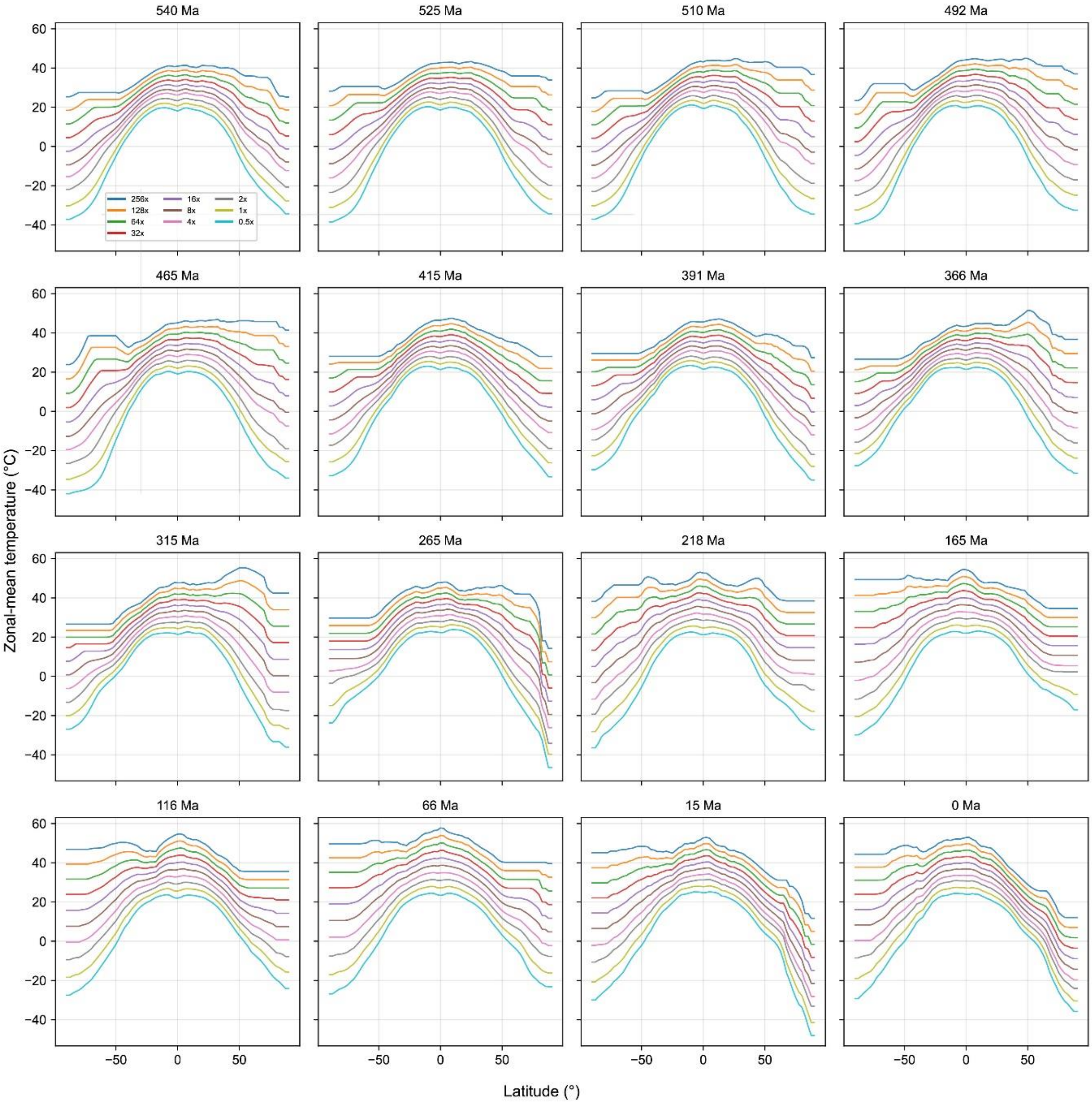

Figure S16. Latitudinal temperature gradients from 2D interpolated HadCM3L simulations after applying a flattening adjustment, across 0.5×–256× PI CO_2 scenarios.

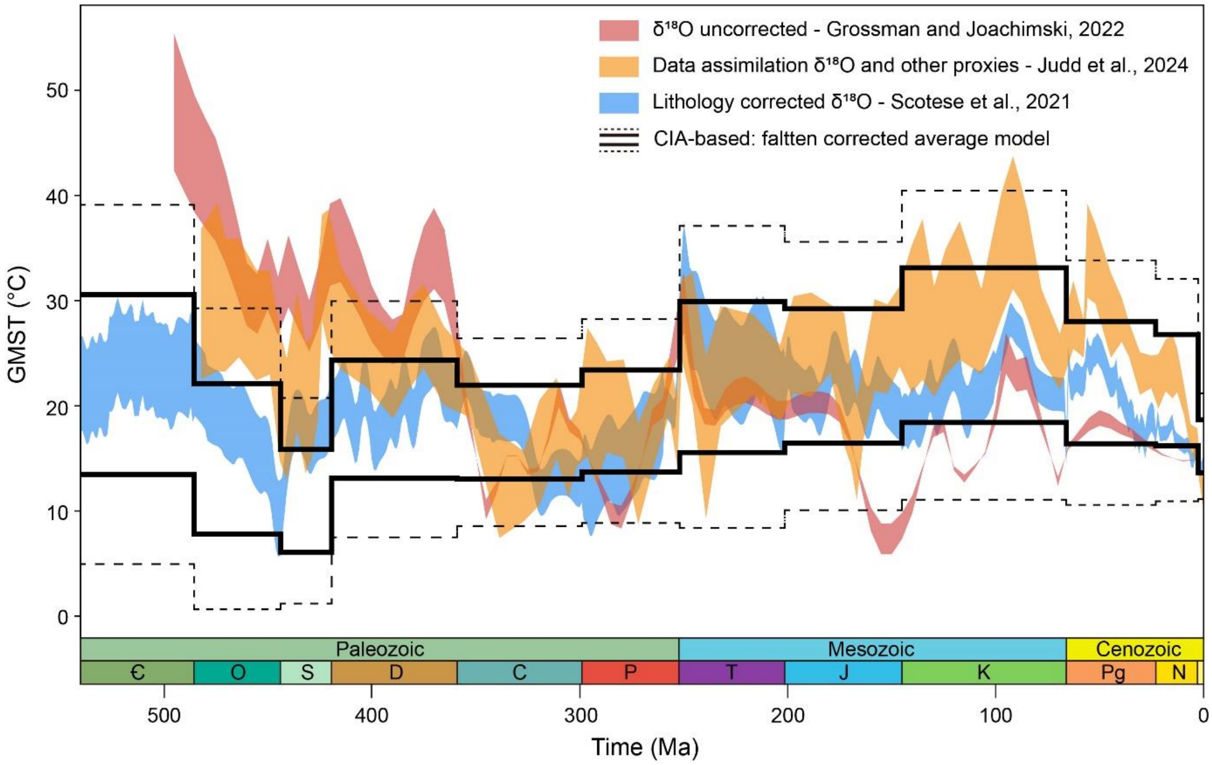

Figure S17. GMST estimates based on HadCM3L simulations after applying the flattening adjustment. Legend as in Fig. S3.

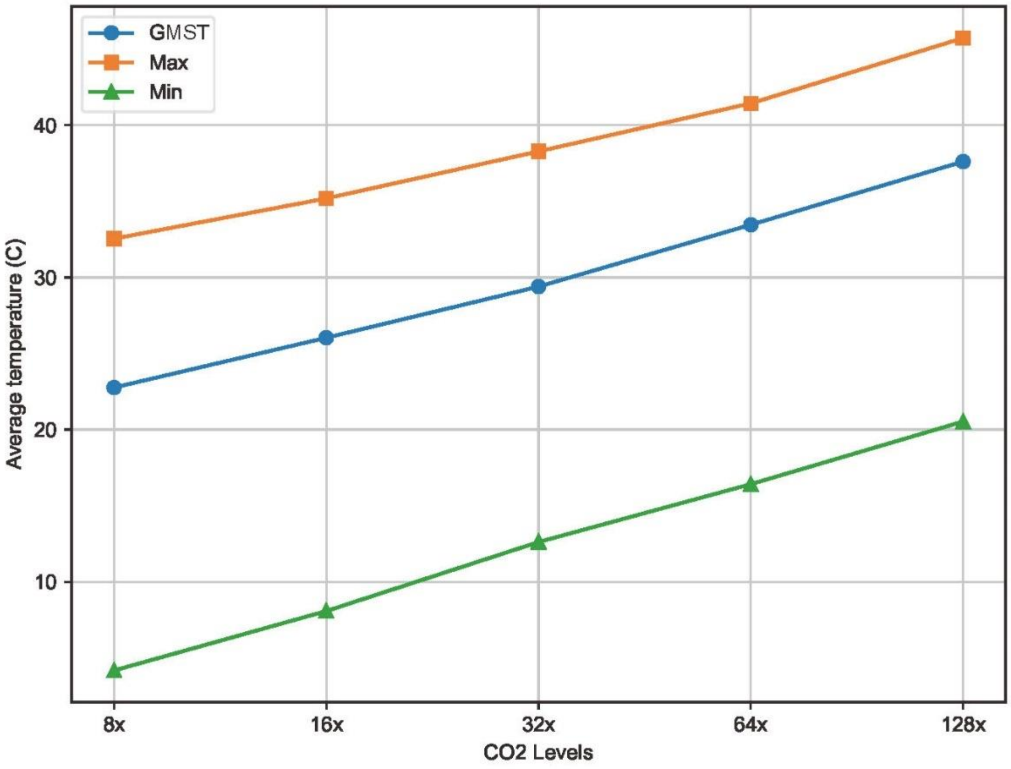

Figure S18. GMST, maximum and minimum temperatures from FOAM simulations at 8×–128× PI CO_2 levels.

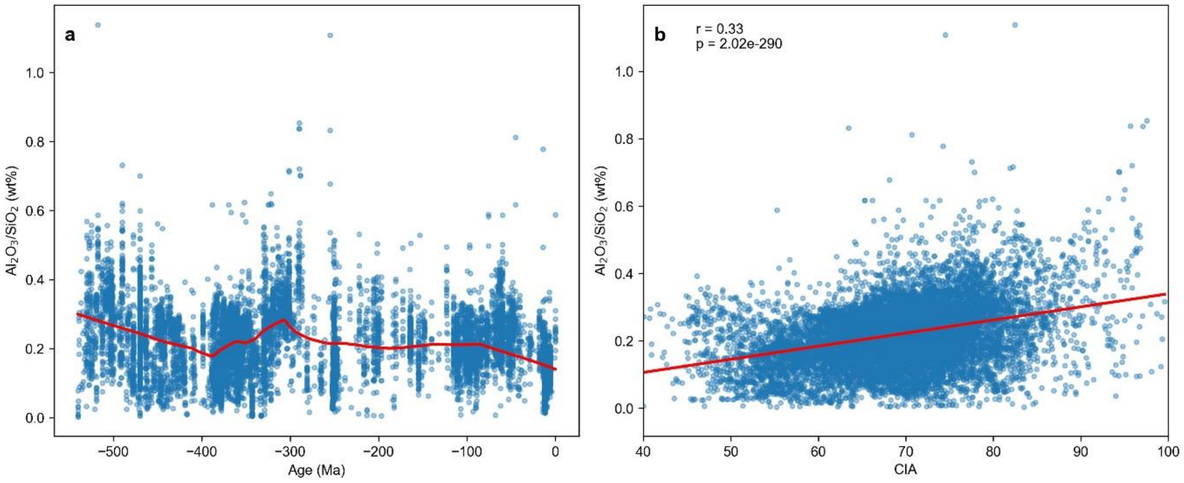

Figure S19. Temporal variation of Al_2O_3/SiO_2 and its correlation with CIA. (a) Temporal variation of the Al_2O_3/SiO_2 ratio across the Phanerozoic. (b) Relationship between Al_2O_3/SiO_2 and CIA values. The red curve in (a) represents a LOWESS regression with a span of 0.1. The red line in (b) shows the linear regression, with a correlation coefficient of $r = 0.33$ ($p < 0.001$).

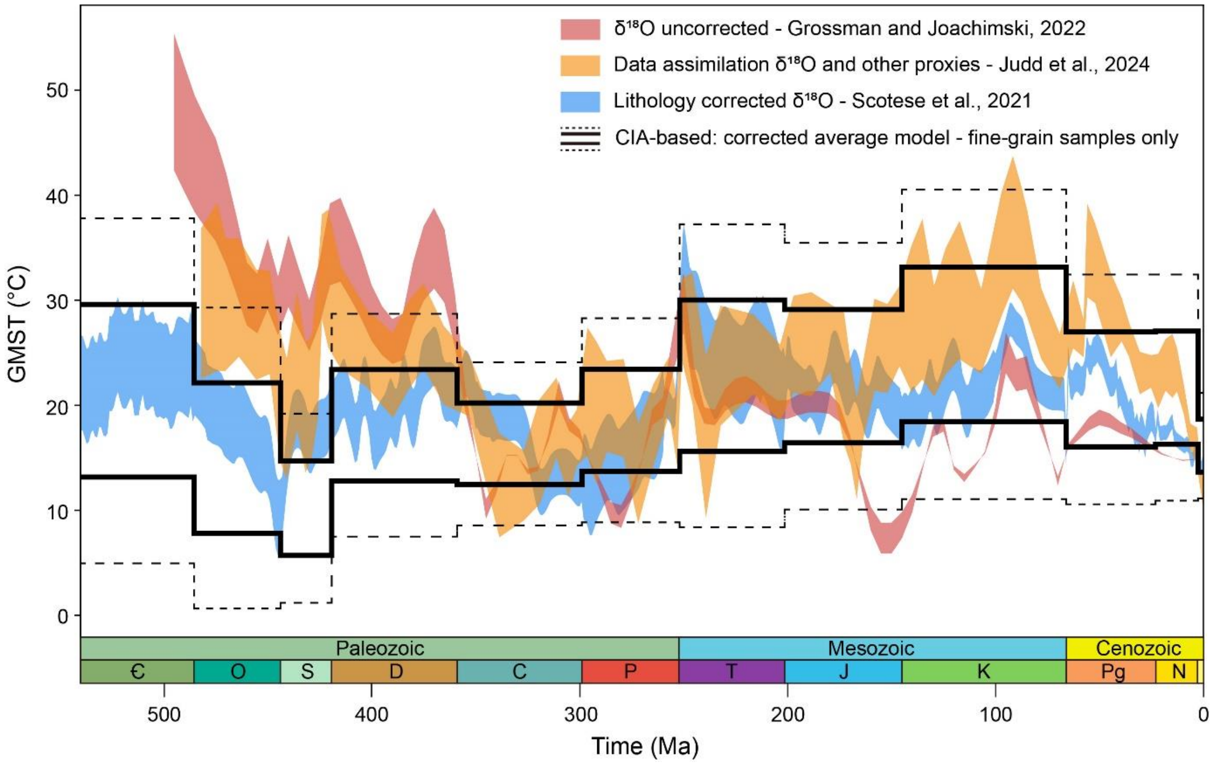

Figure S20. GMST estimates based on samples with $Al_2O_3/SiO_2 \geq 0.1$, representing clay-rich, fine-grained sediments. Legend as in Fig. S3.

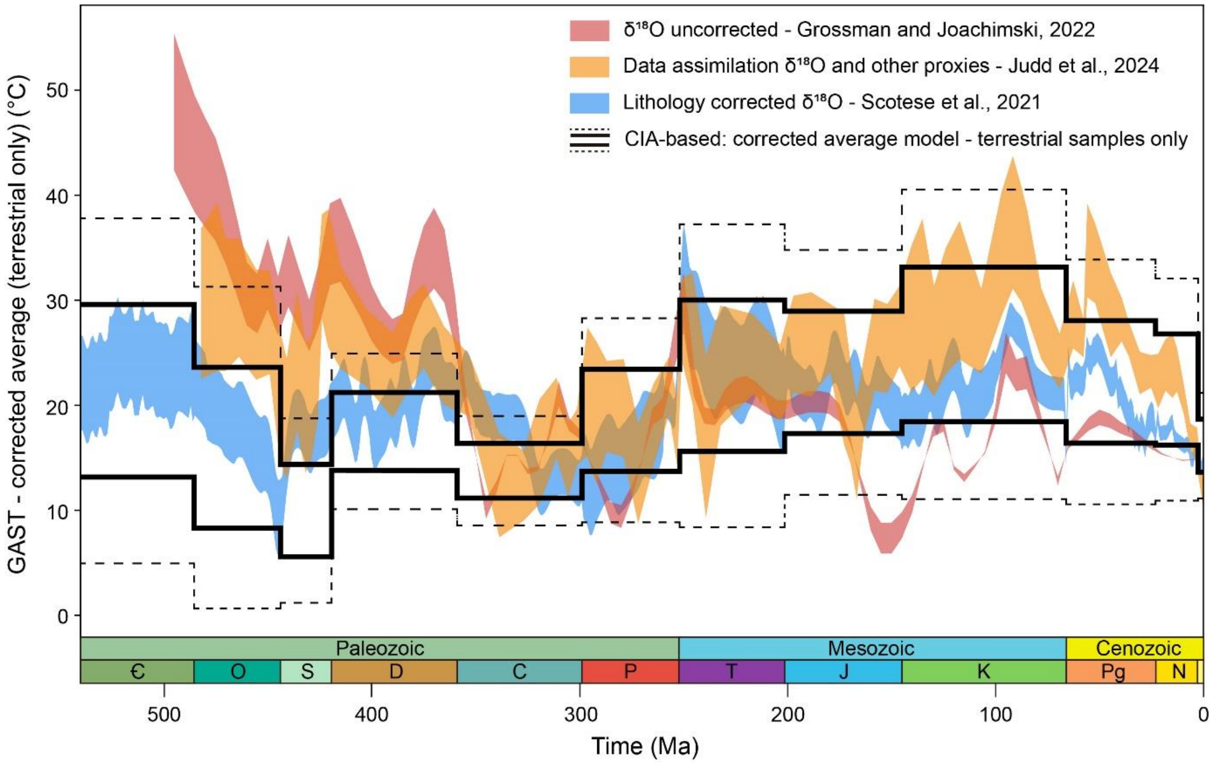

Figure S21. GMST estimates based on terrestrial samples only. A sample is classified as terrestrial if the surrounding 3 × 3 grid cells (approximately 11.25° longitude × 7.5° latitude) are all identified as land in the Scotese paleogeographic maps[52]. Legend as in Fig. S3.

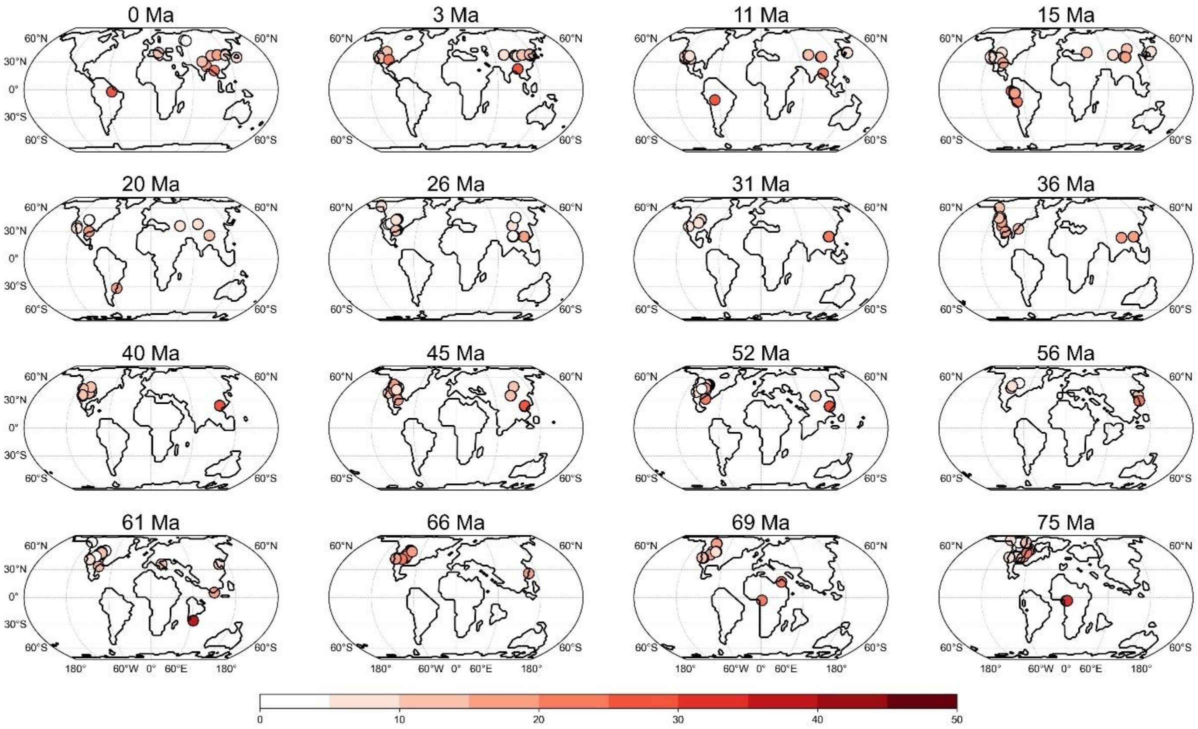

Figure S22. The CIA-inferred temperatures and their geographic distribution from 75 Ma to 0 Ma. The unique sample location is defined as a unique grid cell (2.5° × 3.75°) within HadCM3L. Location symbols are enlarged for visualization purposes, which may result in overlaps on the maps shown in Figs. S22-S28. The colorbar represents temperatures from 0 °C to 50 °C.

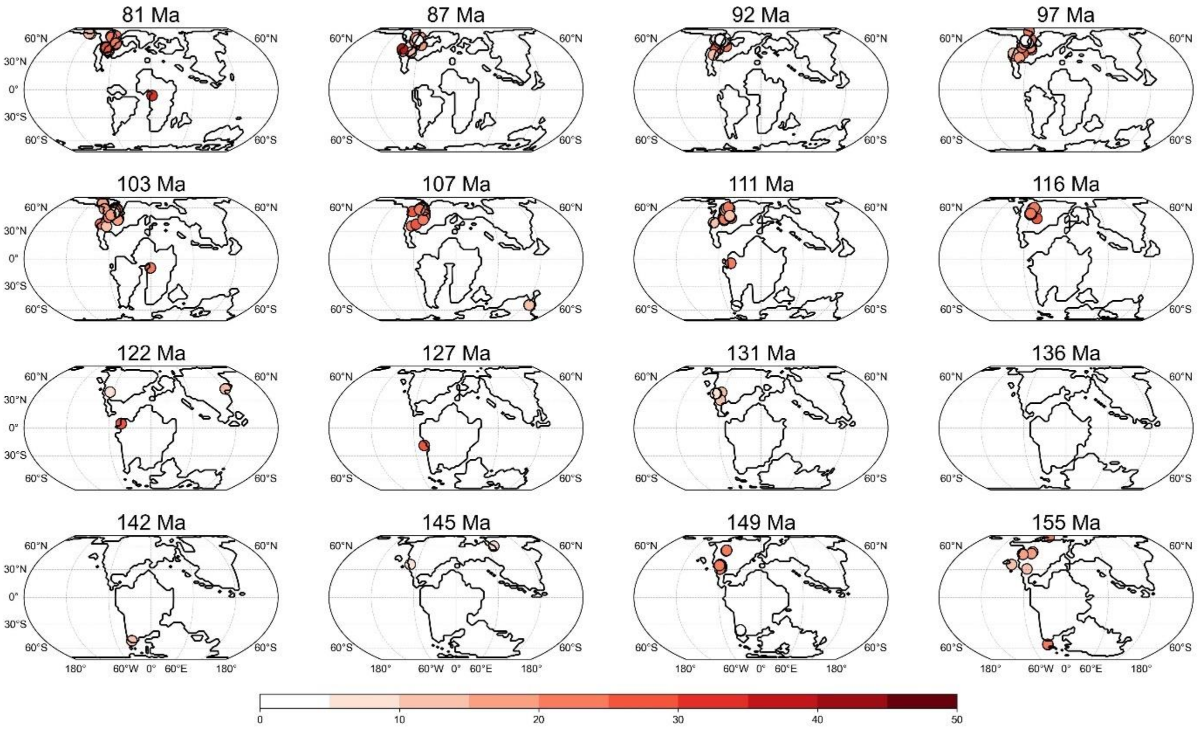

Figure S23. The CIA-inferred temperatures and their geographic distribution from 155 Ma to 81 Ma.

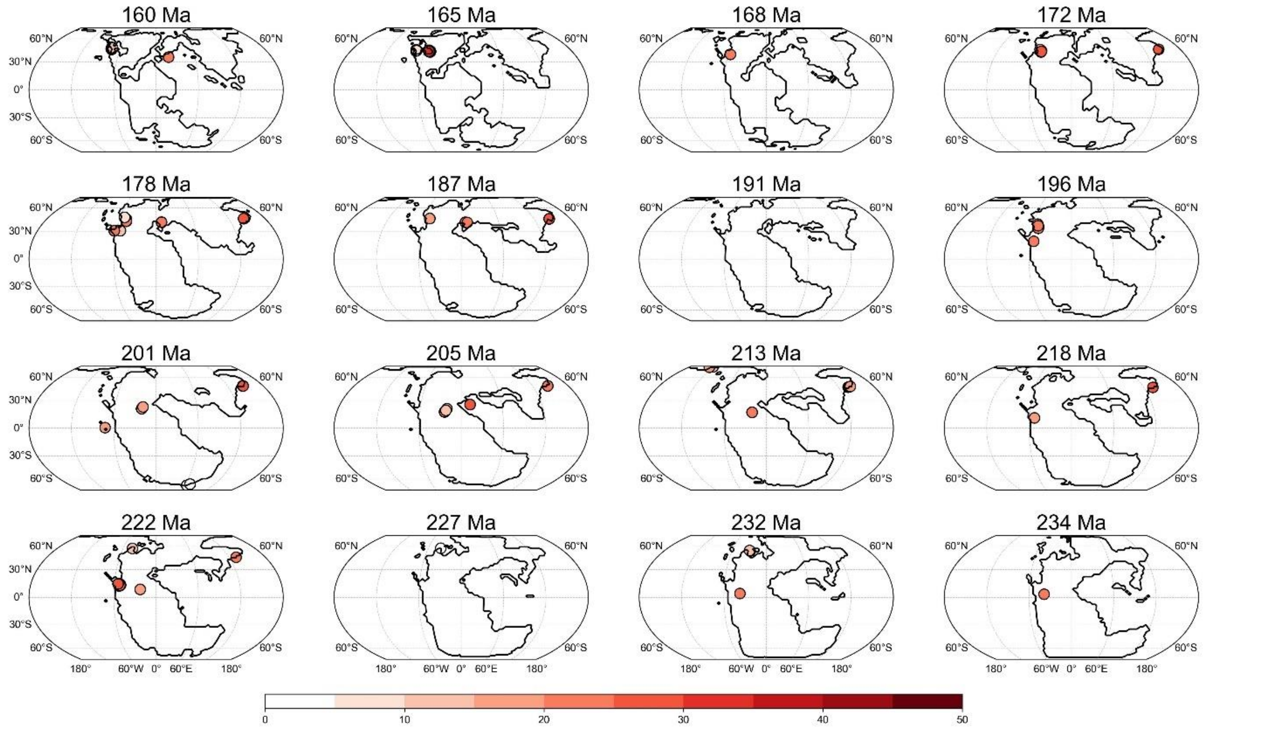

Figure S24. The CIA-inferred temperatures and their geographic distribution from 234 Ma to 160 Ma.

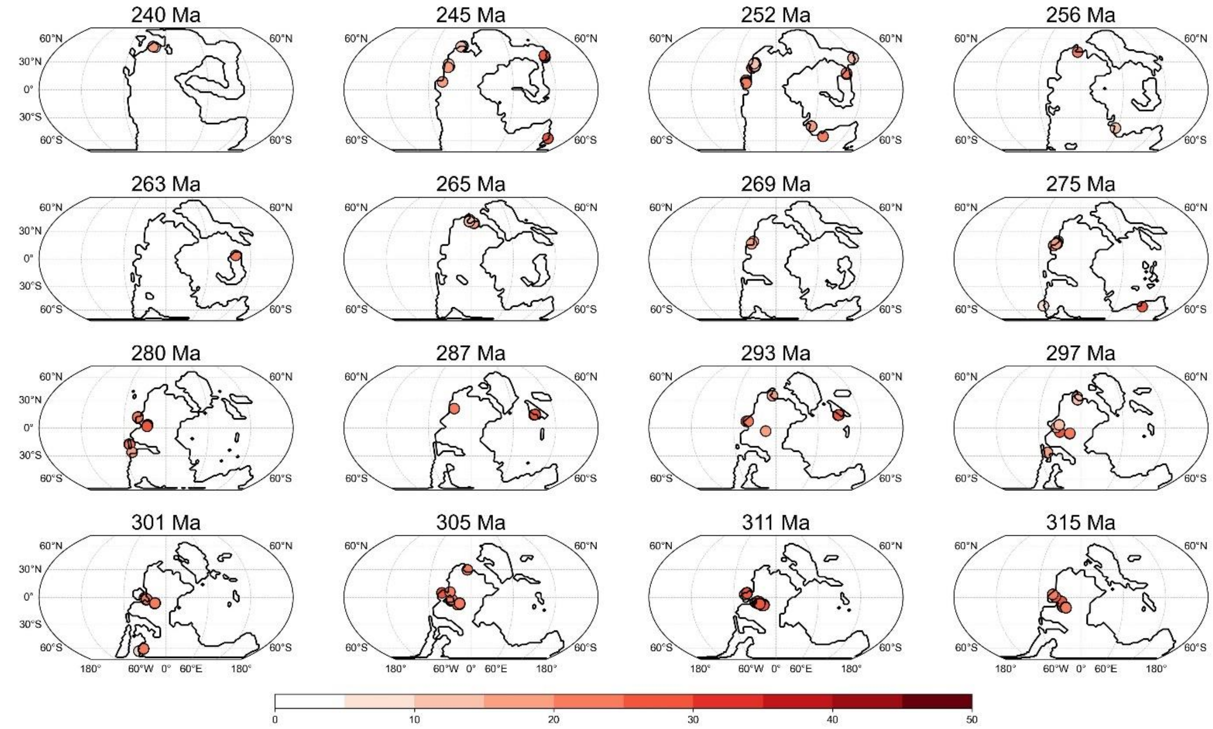

Figure S25. The CIA-inferred temperatures and their geographic distribution from 315 Ma to 240 Ma.

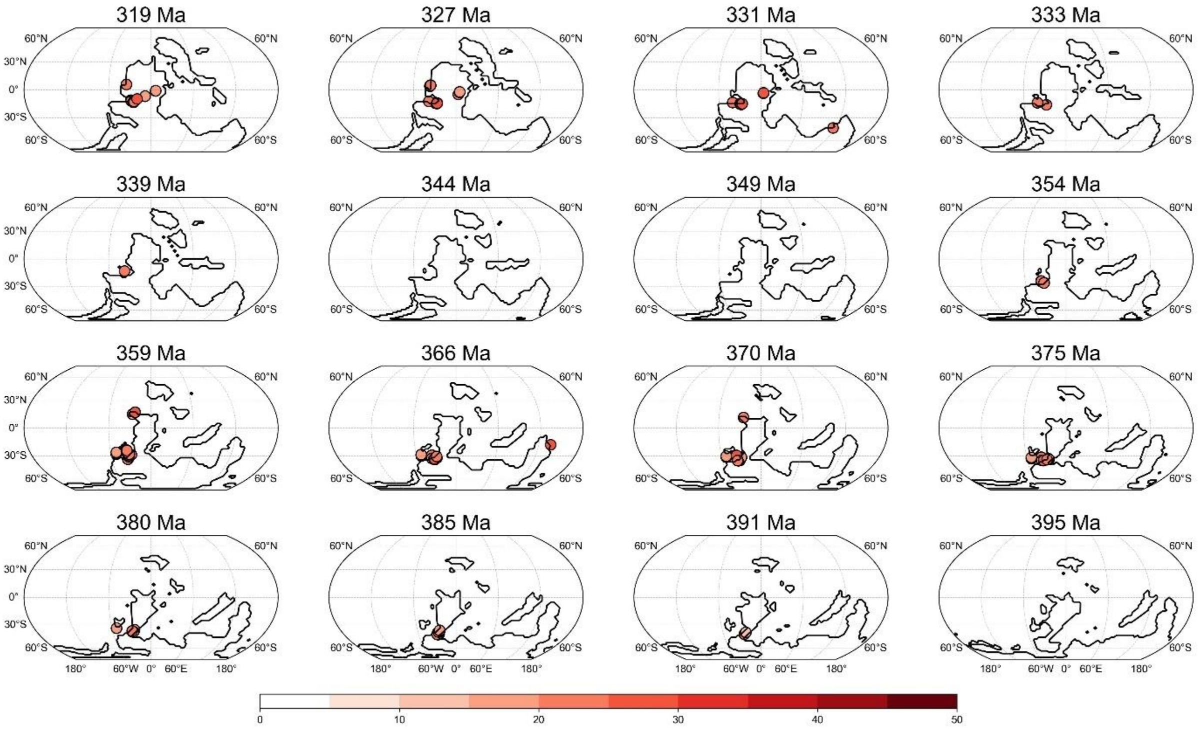

Figure S26. The CIA-inferred temperatures and their geographic distribution from 395 Ma to 319 Ma.

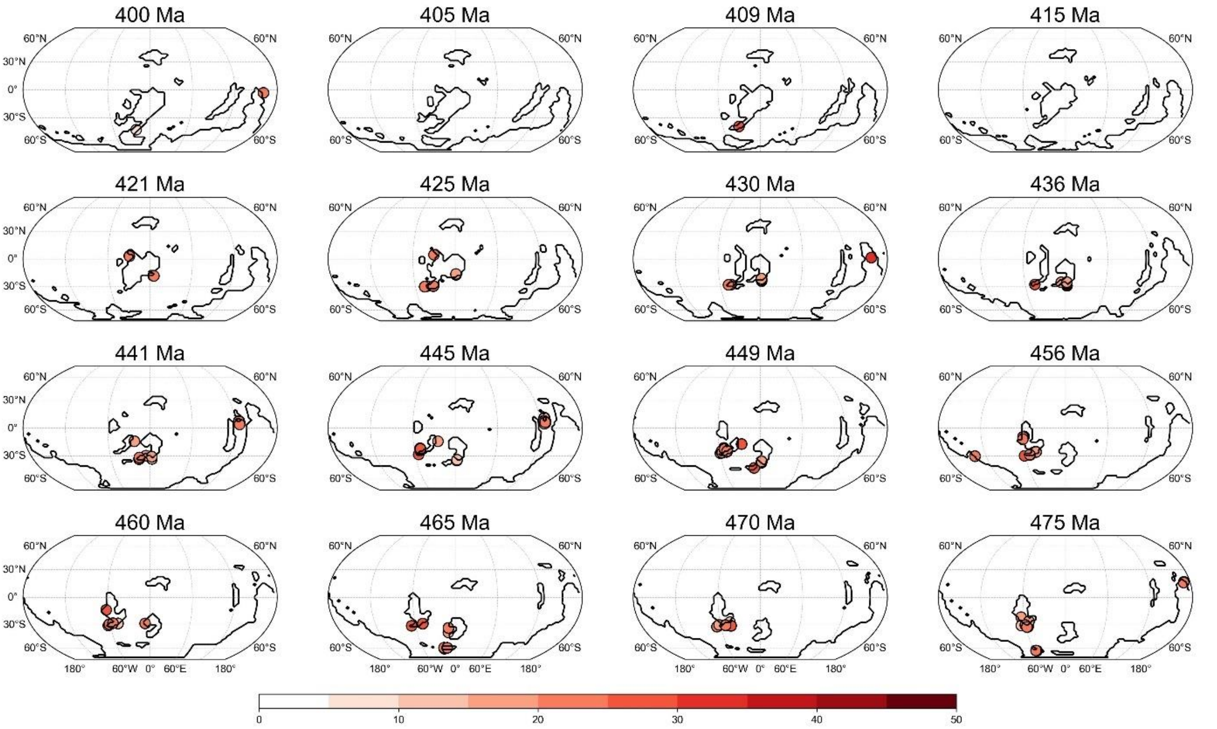

Figure S27. The CIA-inferred temperatures and their geographic distribution from 475 Ma to 400 Ma.

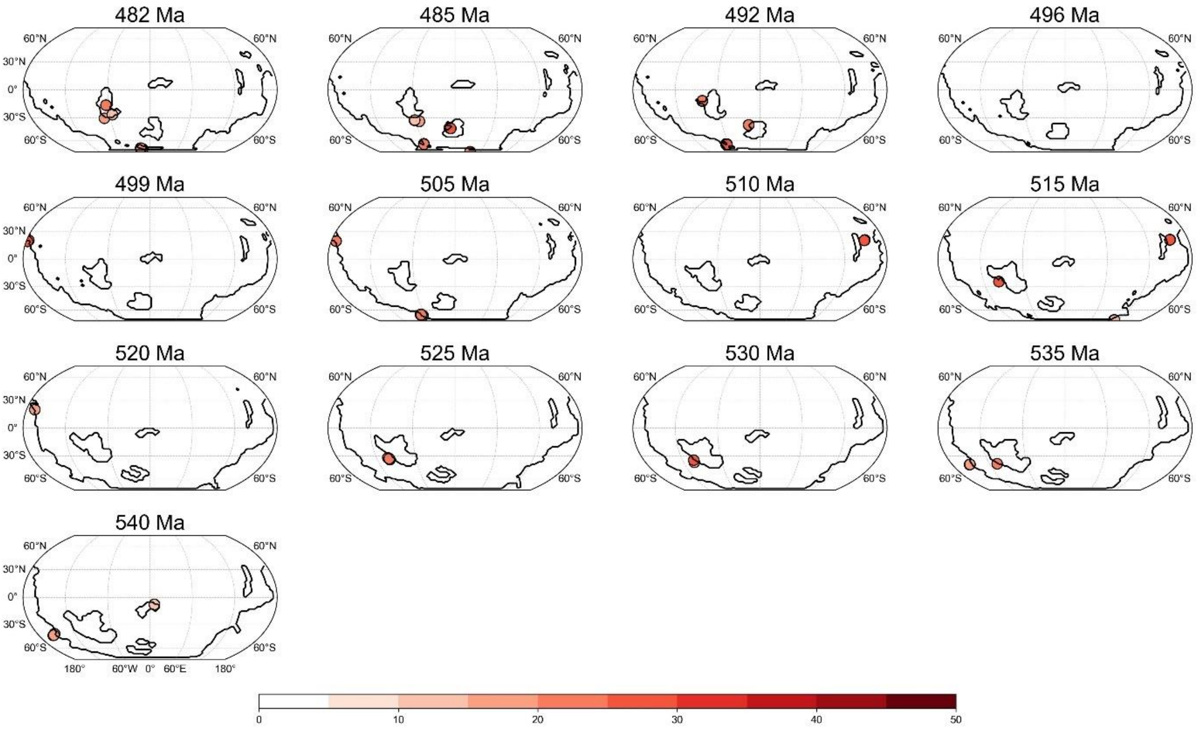

Figure S28. The CIA-inferred temperatures and their geographic distribution from 540 Ma to 482 Ma.
