# Supplementary material for: Tight regulation of Earth’s long-term temperature over Phanerozoic time
Source: Nat Commun. 2026 May 4;17:5995. doi: 10.1038/s41467-026-72672-6 (PMC13347045; doi:10.1038/s41467-026-72672-6)
Supplement: Supplementary file 2 — Description of Additional Supplementary Files [file 41467_2026_72672_MOESM2_ESM.pdf]

Description of Additional Supplementary Files

File name: Supplementary Data 1. Modern river sediment CIA and local temperature

Description: Data S1 contains CIA values and corresponding local temperatures. This dataset is used to generate the linear regression shown in Fig. S6.

File name: Supplementary Data 2. SGP2 dataset and GMST estimates

Description: Data S2 contains the SGP2 and Cenozoic CIA data compilations, as well as GMST estimates for each CIA record based on the data assimilation workflow used in this study.

File name: Supplementary Data 3. GMST at all periods

Description: Data S3 contains GMST estimates and their associated statistics at the period level.

File name: Supplementary Code 1. Python code for GMST estimates

Description: Code S1 contains the Python code used to reproduce the GMST estimates in this study.
