# Supplementary material for: Tight regulation of Earth’s long-term temperature over Phanerozoic time
Source: Nat Commun. 2026 May 4;17:5995. doi: 10.1038/s41467-026-72672-6 (PMC13347045; doi:10.1038/s41467-026-72672-6)
Supplement: Supplementary file 7 — Transparent Peer Review file [file 41467_2026_72672_MOESM7_ESM.pdf]

nature portfolio

Peer Review File

Tight regulation of Earth's long-term temperature over Phanerozoic time

Corresponding Author: Dr Dongyu Zheng

This file contains all reviewer reports in order by version, followed by all author rebuttals in order by version.

Version 0:

Reviewer comments:

Reviewer #1

(Remarks to the Author)
This is an extremely "ambitious" manuscript that attempts to integrate different sedimentary records to piece together the temperature evolution history throughout the entire Phanerozoic. The authors also noted the differences between the CIA in clastic sediments and the seawater $\delta^{18}O$ records: the latter is mixed and uniform on a global scale, making it easy to splice records; whereas the temperature reflected by CIA exhibits regional heterogeneity. Therefore, the authors adopted a stage-by-stage suite of General Circulation paleoclimate model simulations at variable CO_2 levels to upscale regional signals to a global scale.
In fact, modern river catchment studies show that, even at the same location, the CIA values of sediments vary widely, and this variability is mainly controlled by the sediment provenance and grain size. For the modern river sediment datasets, when using a 2°C binning interval, the median CIA values show a good correlation with temperature. This essentially involves averaging through large-scale sampling, which "averages out" other influencing factors such as grain size, rainfall, and vegetation, thereby concluding that temperature controls the degree of weathering in a robust manner. The establishment of this relationship benefits from decades of accumulated massive data from modern river basins, as well as a research approach of "letting nature do the averaging." It can be imagined that if the data volume is insufficient, it would be difficult to obtain such an ideal mathematical relationship.
We observe that in each binning interval, the 25%–75% range of CIA values is approximately 10 units or higher, while the 5%–95% range can reach 40 units or more. Considering that 1 unit of CIA roughly corresponds to 1°C, it is evident that the variability caused by non-temperature factors is enormous. The temperature uncertainty recovered from samples within any single binning interval may cover the entire range of global surface temperature changes throughout the Phanerozoic. Not to mention, the authors further upscale the data to global averages using simulation methods, which undoubtedly introduces new uncertainties.
Returning to the sedimentary records themselves, I believe this dataset is generally modified by diagenesis/metamorphism. Evidence shows that a large number of samples have CIA values below 50 and exhibit a trend of K enrichment (Fig. S22). Under weathering, the evolution of sediments in the A-CN-K ternary diagram should first parallel the CN–A line, then turn toward the A apex, with the turning point's CIA value usually not below 70. If diagenesis is pervasive, then layers with stronger diagenetic alteration will have lower CIA values, and the reconstructed paleotemperatures will also be biased low, which may explain certain stages where reconstructed temperatures are significantly low. I suggest that the authors could further clean the data using some geochemical indicators or diagrammatic methods, for example, referencing the recent review paper by Thomas J. Algeo published in Chemical Geology.
In terms of model calculations, I am not an expert and cannot offer specific suggestions. However, I believe the main source of error in this paper may lie in the sedimentary records themselves and the uncertainty of the CIA-temperature conversion equation, and the authors should enhance the rigor of the study in these two aspects. I believe the authors can accept that the reconstructed temperature results have large uncertainties, but the major warming and cooling trends in geological historical periods should be reflected. Currently, the temporal resolution of the reconstruction results is low, with the entire Phanerozoic possibly showing only about ten change nodes, and such precision may be insufficient to support the application of this paper's results to research in other fields.
In summary, I believe our attitude toward this reconstructed record should be cautious. I suggest that the authors conduct some tests on the reconstruction results, for example, trying to use a period with well-constrained paleotemperatures—such as the Cenozoic cooling trend—to verify the temperature evolution pattern proposed in this paper.

(Remarks on code availability)

Reviewer #2

(Remarks to the Author)
Review Report on Manuscript NCOMMS-25-84283
This manuscript presents a comprehensive reconstruction of Phanerozoic global temperatures using the CIA from a large global database of siliciclastic sedimentary rocks. By integrating this proxy with paleoclimate model simulations, this study provides an independent estimate of Global Average Surface Temperature (GAST) that is independent of oxygen isotope records. The study challenges the conventional view of an extremely hot early Paleozoic and instead supports a more stable and tightly regulated climate system throughout the Phanerozoic. The approach is innovative and the dataset is impressive, though several issues require further clarification and discussion.

Major Comments:

1. CIA-Derived Temperature Uncertainties:
While the CIA proxy offers broader spatial coverage in geological history, compared to oxygen isotopes, the associated GAST uncertainties are substantial (ranging up to 15–30°C in Figure 2). Could the authors propose potential strategies to reduce these uncertainties in future work? For instance, might refined statistical methods or additional proxies help constrain these error margins?

2. Impact of Land Plant Evolution on CIA:
The influence of vascular plant colonization on weathering regimes and CIA values requires more thorough discussion. The application of modern CIA-temperature relationships to pre-vegetation periods (e.g., early Paleozoic) needs stronger justification. Although references 37 and 38 are cited, other non-temperature factors (e.g., the potential effects of soil calcification in ref 38) should be explicitly addressed.

3. Data Cleaning and Selection:
The manuscript lacks a detailed description of the data cleaning process. Specifically, how were issues such as K-metasomatism (A-CN-K trends), and the effects of aridity (e.g., during the Triassic) addressed?

4. Model Extrapolation and CO_2 Forcing:
While I am not a specialist in the model used here, I have a question regarding the simulated climate under different CO_2 levels. I am particularly curious about the physical mechanism behind the apparent inverse relationship between temperature and the latitudinal temperature gradient at high CO2 concentrations (in Figure S9). Additionally, it would be very helpful if the CO_2 values used for each stage could be indicated directly in the relevant figures. Correlating these chosen values with CO_2 levels reconstructed by other methods would further strengthen the justification for the parameter selection.

Minor Comments:

Line 50: multicellular organisms in the Cambrian-Ordovician “,” the colonization of land by plants in the Ordovician-Devonian

Lines 95–97: Expand on how the expansion of vascular plants is reflected in the weathering intensity and aluminum mobility records.

Lines 122–129: Address potential latitudinal biases, especially given the Cenozoic data's reliance on North America. Can additional regional data be incorporated?

Line 137: Provide a detailed description of the data cleaning methodology.

Lines 198–202: Explain the higher GAST values during the P-C derived from CIA compared to oxygen isotopes (in figure2). If aridity is invoked for the Triassic, what explains the P–C discrepancy?

Lines 611–619: Consider displaying the CO_2 values used in each stage and compare them with other published reconstructions.

Lines 768–769: Discuss the implications of A-CN-K trends indicating potassium metasomatism. How was diagenetic alteration accounted for, particularly given that K-metasomatism may depress CIA values and lead to underestimated temperatures?

Figure S9: Explain the occurrence of inverted latitudinal temperature gradients between 269–315 Ma, particularly starting at 4–8×CO_2.

(Remarks on code availability)

Reviewer #3

(Remarks to the Author)
I like this paper a lot. It's great to have estimates of Phanerozoic temperatures independent of oxygen isotopes, especially those based on a well-established method like CIA. As I read the paper, I had many questions, but all were answered by the methods. The extensive methods are well described and seemingly address all caveats that I could think of. My comments are minor.
There are a few grammatical issues, mostly missing commas, that should be cleaned up. I pointed some of them out.
Line 50: comma needed.
Line 111: comma needed.
Line 60: I've always found these temperature estimates to be dubious.
Line 144 through 148: This sentence needs to be two—too much information for 1.
Line 149: comma needed.
Line 164: comma needed.
Lines 199 through 202: I see what you are saying about the Triassic, but I also think the Permian should be mentioned as a potential outlier. Your Permian estimate is below the other curves and the general trend of lower temperatures in Permian and warmer in the Triassic appears to be reversed in your dataset. Why? Even at the rough resolution of your dataset, doesn't that seem like a trend that would be preserved? Is it sampling? Are the bins biased by an overabundance of P-T boundary data?
Equation 1: There is an extra space in CaO*
Equation 2: I think there are missing parentheses.
Line 590: Shouldn't there be a last name to go with citation number 69? If not, shouldn't the number be non-superscript and separated from the preceding word? There are several others like that, and they should all be changed for readability.
Figure S4 – 5: It would be more convenient to put the last name of the model author in the figure caption instead of a number.
Figure S15 – 20: I like these figures, but the individual maps are way too small to be properly assessed. They all need to be bigger.

(Remarks on code availability)

Version 1:

Reviewer comments:

Reviewer #1

(Remarks to the Author)
I am pleased to see that the authors have conducted a thorough revision; however, there are several critical issues I would like to further discuss:

While the sample size has increased significantly, in deep-time (especially Paleozoic) studies, sample quantity does not equate to spatial representativeness. I suggest that the authors provide a paleogeographic distribution map of the data points to demonstrate that the expanded dataset offers sufficient coverage across different paleolatitudes and tectonic blocks, rather than being a mere mechanical increase in data volume.

The CIA-temperature conversion equation may exhibit a non-linear response under extreme cold or hot conditions. For instance, in hyper-greenhouse climates, CIA values may exceed 90, leading to a "saturation effect" (where the proxy becomes insensitive to further temperature increases). Conversely, in extreme icehouse conditions, physical erosion dominates, and the chemical weathering signal might be completely obscured. I recommend that the authors briefly discuss the applicability of the equation in these end-member states or acknowledge the potential for larger non-linear errors at temperature extremes, while emphasizing the reliability of the results in capturing first-order patterns.

The authors utilize the average Upper Continental Crust (UCC) as a protolith proxy for potassium correction. Given the immense geological timescale of this study, variations in UCC composition over different periods could lead to substantial calculation biases. I suggest that the authors conduct a Sensitivity Analysis to verify whether fluctuations in parent rock composition might compromise the stability of the reconstructed Phanerozoic first-order temperature evolution trends.

Regarding the grain-size effect, the authors' mention of "selecting fine-grained siliciclastic rocks" remains a qualitative treatment. In deep-time records, it is inherently difficult to completely exclude the dilution effect of silt on the clay fraction. The authors should, at the very least, verify whether the data sources include marine strata, considering the potential presence of authigenic clays formed through processes such as reverse weathering in marine environments. Furthermore, is it possible to utilize geochemical indicators (e.g., elemental ratios) or statistical methods to quantitatively evaluate the influence of sedimentary processes, such as grain-size sorting, on the weathering signals?

(Remarks on code availability)

Reviewer #2

(Remarks to the Author)
I have carefully read the authors' response and the revised manuscript. I believe the authors have addressed the reviewers' comments and provided a thorough response, particularly regarding their efforts to evaluate the local signals of the CIA and the effects of K-metasomatism during data cleaning and processing.This study presents a novel alternative method for paleotemperature reconstruction using big data and is worthy of publication.

(Remarks on code availability)

Version 2:

Reviewer comments:

Reviewer #1

(Remarks to the Author)
I am pleased with the authors' quick and comprehensive response to the feedback. The manuscript is now in excellent shape. I am confident that this work will contribute meaningfully to the study of deep-time environments and geochemistry. While many questions in this field warrant further discussion, there is clearly much more to be unraveled from the siliciclastic record.

(Remarks on code availability)

Open Access This Peer Review File is licensed under a Creative Commons Attribution 4.0 International License, which permits use, sharing, adaptation, distribution and reproduction in any medium or format, as long as you give appropriate credit to the original author(s) and the source, provide a link to the Creative Commons license, and indicate if changes were made.
In cases where reviewers are anonymous, credit should be given to 'Anonymous Referee' and the source.
The images or other third party material in this Peer Review File are included in the article's Creative Commons license, unless indicated otherwise in a credit line to the material. If material is not included in the article's Creative Commons license and your intended use is not permitted by statutory regulation or exceeds the permitted use, you will need to obtain permission directly from the copyright holder.
To view a copy of this license, visit https://creativecommons.org/licenses/by/4.0/

Responses to reviewers:

Tight regulation of Earth's long-term temperature over Phanerozoic time

Dongyu Zheng, Alex G. Lipp, Alexander Farnsworth, Shufeng Li, Andrew S. Merdith, Khushboo Gurung, Mingcai Hou, Anqing Chen, Zixi Hou, Daniel J. Lunt, Erik A. Sperling, Paul J. Valdes, Benjamin J. W. Mills

The reviewers' comments are in black, and our responses in blue. Line numbers refer to the clean version of the revised manuscript.

We thank the reviewers for their insightful comments and have addressed all the points raised. In this revision:

- We updated the CIA dataset with the most recent SGP Phase 2 data and with additional Cenozoic records from the literature, making 18,368 records in total, ~1.7x size of the original dataset.
- We added several sections that explicitly describe our data filtering procedures, and have added a K-metasomatism correction and a further method validation using Cenozoic records.
- We substantially revised the manuscript text and added additional figures to both the main text and supplementary materials to clarify the methodology and improve readability.

Together, the expanded dataset and refined analysis have improved our reconstruction of the major warming and cooling trends over the Phanerozoic. These revisions further reinforce our conclusions that early Paleozoic GMST was comparable to later warm periods, in contrast to previous work suggesting that it was anomalously hot.

Reviewer #1 (Remarks to the Author):

This is an extremely "ambitious" manuscript that attempts to integrate different sedimentary records to piece together the temperature evolution history throughout the entire Phanerozoic. The authors also noted the differences between the CIA in clastic sediments and the seawater $\delta^{18}O$ records: the latter is mixed and uniform on a global scale, making it easy to splice records; whereas the temperature reflected by CIA exhibits regional heterogeneity. Therefore, the authors adopted a stage-by-stage suite

of General Circulation paleoclimate model simulations at variable CO_2 levels to upscale regional signals to a global scale. In fact, modern river catchment studies show that, even at the same location, the CIA values of sediments vary widely, and this variability is mainly controlled by the sediment provenance and grain size. For the modern river sediment datasets, when using a 2°C binning interval, the median CIA values show a good correlation with temperature. This essentially involves averaging through large-scale sampling, which "averages out" other influencing factors such as grain size, rainfall, and vegetation, thereby concluding that temperature controls the degree of weathering in a robust manner. The establishment of this relationship benefits from decades of accumulated massive data from modern river basins, as well as a research approach of "letting nature do the averaging." It can be imagined that if the data volume is insufficient, it would be difficult to obtain such an ideal mathematical relationship. We observe that in each binning interval, the 25%–75% range of CIA values is approximately 10 units or higher, while the 5%–95% range can reach 40 units or more. Considering that 1 unit of CIA roughly corresponds to 1°C, it is evident that the variability caused by non-temperature factors is enormous. The temperature uncertainty recovered from samples within any single binning interval may cover the entire range of global surface temperature changes throughout the Phanerozoic. Not to mention, the authors further upscale the data to global averages using simulation methods, which undoubtedly introduces new uncertainties.

R: We fully agree that CIA values exhibit substantial local variability, and that the robustness of the modern CIA–temperature relationship emerges only when very large datasets are aggregated, allowing other influences to be effectively averaged out. Our deep-time approach is motivated by this same principle. Because Phanerozoic sedimentary records are inherently heterogeneous and spatially incomplete, we rely on (i) large-scale spatial aggregation of CIA data at Period level and (ii) stage-specific General Circulation Model simulations to upscale regional CIA–temperature relationships to the global scale.

We explicitly acknowledge that this approach carries uncertainties in absolute temperature estimates, particularly given the limited and uneven spatial coverage of deep-time data. However, our primary conclusions are based on first-order temporal patterns and temperature envelopes rather than precise absolute values. Particularly, we

show that while our uncertainty window is indeed large, previous extremely warm climate interpretations based on oxygen isotopes still plot outside of it, allowing us to effectively challenge them.

In the revision we have made sure these uncertainties are clearly stated (lines 88-108), and have substantially enlarged the CIA dataset (~1.7x of the previous sample size; lines 573-587) and added further sensitivity tests, including data screening strategies, K-metasomatism corrections, and different CO_2 forcing scenarios (lines 589-648; lines 191-204), which demonstrate that these uncertainties do not alter the central conclusion of this study: early Paleozoic global average surface temperatures were comparable to those of later Phanerozoic warm periods, and were not exceptionally high. Thus, while uncertainties remain in the exact magnitude of reconstructed temperatures, we do not believe that they affect the robustness of our main conclusions. We have also made it clearer in the revision that current oxygen isotope methods do suffer from similar issues: Paleozoic records are from shallow water species which also record only regional conditions and have key non-temperature dependencies such as salinity and critically the seawater oxygen isotope composition – which is very poorly understood (lines 101-108).

Returning to the sedimentary records themselves, I believe this dataset is generally modified by diagenesis/metamorphism. Evidence shows that a large number of samples have CIA values below 50 and exhibit a trend of K enrichment (Fig. S22). Under weathering, the evolution of sediments in the A-CN-K ternary diagram should first parallel the CN–A line, then turn toward the A apex, with the turning point's CIA value usually not below 70. If diagenesis is pervasive, then layers with stronger diagenetic alteration will have lower CIA values, and the reconstructed paleotemperatures will also be biased low, which may explain certain stages where reconstructed temperatures are significantly low. I suggest that the authors could further clean the data using some geochemical indicators or diagrammatic methods, for example, referencing the recent review paper by Thomas J. Algeo published in Chemical Geology.

R: We appreciate this insightful comment regarding diagenetic modification and its potential impact on CIA-based temperature reconstructions. We agree that pervasive

diagenesis, particularly K-metasomatism, can depress CIA values and bias reconstructed temperatures toward unrealistically low values.

Following the reviewer's suggestion, we carefully re-examined our dataset in light of the recent synthesis by Algeo et al. (2025) and implemented an explicit K-metasomatism correction (lines 614-647). In the revised manuscript, we apply the correction framework of Fedo et al. (1995) using the formulation of Panahi et al. (2000), which adjusts K_2O to recover pre-metasomatic compositions. This method requires the molar fraction of K_2O in the unaltered parent material. Because protolith compositions are unknown for most samples in our global deep-time compilation, we adopt the average Upper Continental Crust (UCC) composition as a proxy (Al_2O_3 = 15.4 wt%, Na_2O = 3.28 wt%, CaO = 3.60 wt%, K_2O = 2.81 wt%; Rudnick et al. 2005), yielding a parent-material molar K fraction of ~0.10.

After applying the Panahi correction, we compared measured and corrected K_2O values and retained the smaller of the two, as the corrected value represents the inferred pre-metasomatic composition and should not exceed the measured value if K addition occurred. Cases where the correction yields higher K_2O values are interpreted as artefacts of applying a generalized UCC protolith to locally variable source rocks and are therefore not considered physically meaningful. This treatment is consistent with Algeo et al. (2025), who emphasize that K-metasomatism can follow multiple alteration vectors, and that the pure-addition endmember assumed by the Fedo–Panahi correction likely overestimates K enrichment in many natural systems where partial replacement of CaO+Na_2O dominates.

As a result, the K-corrected CIA values should be interpreted as upper-bound estimates, whereas uncorrected CIA values represent the lower-bound case in which diagenetic effects are minimal. We therefore use the corrected CIA values for our primary GMST reconstruction, while explicitly acknowledging that the true values likely lie between the corrected and uncorrected endmembers.

Applying the K-metasomatism correction brings the median (25–50th percentile) estimates into closer agreement with known icehouse-greenhouse cycles (Fig. 3a). And

our central conclusion remains unchanged: early Paleozoic GMST values are not likely to be higher than those of later Phanerozoic warm periods.

In terms of model calculations, I am not an expert and cannot offer specific suggestions. However, I believe the main source of error in this paper may lie in the sedimentary records themselves and the uncertainty of the CIA-temperature conversion equation, and the authors should enhance the rigor of the study in these two aspects. I believe the authors can accept that the reconstructed temperature results have large uncertainties, but the major warming and cooling trends in geological historical periods should be reflected. Currently, the temporal resolution of the reconstruction results is low, with the entire Phanerozoic possibly showing only about ten change nodes, and such precision may be insufficient to support the application of this paper's results to research in other fields.

R: As above, we agree with the reviewer about the key sources of uncertainty in our reconstruction. In our revision we have been more careful to explicitly acknowledge these uncertainties and have taken several steps as suggested to improve the rigor and robustness of the reconstruction. We significantly expanded the CIA dataset, incorporating the recently published SGP Phase 2 database (Farrell et al., 2026) and compiled additional Cenozoic CIA data from 36 peer-reviewed studies. We further applied stricter data-cleaning criteria, including selecting only fine-grained siliciclastic rocks, removing samples with CaO > 8 wt% to minimize carbonate contamination (Lipp et al., 2021; Millikin et al., 2025), and excluding documented recycled rocks and statistical outliers beyond 1.5× the interquartile range (IQR; i.e., below Q1 − 1.5×IQR or above Q3 + 1.5×IQR). After this data cleaning, the expanded dataset contains 18,368 records, approximately ~1.7x the size of the previous dataset. In addition, we applied established K-metasomatism corrections following Fedo et al. (1995) and Algeo et al. (2025) to reduce the influence of diagenetic modification on CIA values (lines 573-647).

We are pleased to see that this improved dataset and analysis has improved our reconstruction of the major warming and cooling trends over the Phanerozoic. We capture generally-agreed cooling between the early Cambrian and Ordovician-Silurian boundary, we also capture the Devonian warming and slight cooling into the

Carboniferous, the Mesozoic and Early Cenozoic hothouses, and cooling from the Neogene to present (Fig. 3a).

As a further test of our method and its wider application, we have also now plotted an expanded comparison of our method over the Cenozoic at the Epoch level, where temperature evolution is relatively well constrained from benthic foraminiferal $\delta^{18}O$ (Westerhold et al., 2020) and CIA data is reasonably abundant at 4,788 records. Our results successfully reproduce the major features of Cenozoic climate evolution, and the CO_2 scenarios in our GCM that best fit the CIA-derived temperatures are consistent with independent proxy-based CO_2 reconstructions for the Cenozoic (CenCO2PIP, 2023). This validation provides confidence that, despite uncertainties in individual CIA values, the approach can capture first-order temperature trends providing sufficient CIA data is available (lines 191-204; Figure 4).

Reviewer #2 (Remarks to the Author):

Review Report on Manuscript NCOMMS-25-84283

This manuscript presents a comprehensive reconstruction of Phanerozoic global temperatures using the CIA from a large global database of siliciclastic sedimentary rocks. By integrating this proxy with paleoclimate model simulations, this study provides an independent estimate of Global Average Surface Temperature (GMST) that is independent of oxygen isotope records. The study challenges the conventional view of an extremely hot early Paleozoic and instead supports a more stable and tightly regulated climate system throughout the Phanerozoic. The approach is innovative and the dataset is impressive, though several issues require further clarification and discussion.

Major Comments:

1. CIA-Derived Temperature Uncertainties:

While the CIA proxy offers broader spatial coverage in geological history, compared to oxygen isotopes, the associated GMST uncertainties are substantial (ranging up to

15–30°C in Figure 2). Could the authors propose potential strategies to reduce these uncertainties in future work? For instance, might refined statistical methods or additional proxies help constrain these error margins?

R: We agree that CIA-based GMST reconstructions carry substantial uncertainties, arising both from limited spatial coverage in deep time and from local non-climatic controls on CIA values, and that reducing these uncertainties is an important direction for future work.

In the revision we have introduced and applied several methods to reduce uncertainty and increase robustness of the method. We have significantly increased the number of samples in the dataset to better average out local non-temperature effects, while also introducing quality control methods to address diagenesis and carbonate contamination. These methods significantly improve the fit of our reconstruction to greenhouse-icehouse cycles over the Phanerozoic, and we show that applying our approach to the Cenozoic using more than 4,788 CIA records at the epoch level now successfully captures major features of climate evolution (lines 191-204; Fig. 4). This test suggests that increased data density, with suitable correction and quality control will yield better Phanerozoic records in the future.

In addition, integrating complementary paleoclimate proxies offers a promising avenue to further constrain GMST estimates. Combining CIA with lithological indicators, biological proxies, and isotopic records would allow cross-validation among independent climate signals and help narrow uncertainty ranges. More advanced statistical frameworks, such as multi-proxy data assimilation or Bayesian approaches, could also explicitly reduce uncertainties and optimally weight different proxies according to their strengths and limitations. We have added a section on further steps in the revision (lines 811-829).

2. Impact of Land Plant Evolution on CIA:

The influence of vascular plant colonization on weathering regimes and CIA values requires more thorough discussion. The application of modern CIA-temperature relationships to pre-vegetation periods (e.g., early Paleozoic) needs stronger

justification. Although references 37 and 38 are cited, other non-temperature factors (e.g., the potential effects of soil calcification in ref 38) should be explicitly addressed.

R: This is an important point. We agree that applying modern CIA–temperature relationships to pre-vegetation intervals requires careful justification and explicit consideration of non-temperature controls.

Vegetation can influence weathering by enhancing mineral dissolution through deeper rooting systems, organic acid production, and increased soil CO_2 (e.g., Berner et al., 1997). However, multiple lines of evidence suggest that the first-order intensity of chemical weathering did not undergo a stepwise increase coincident with the Paleozoic expansion of vascular plants. In particular, the Al/Ti ratio in paleosols shows relative stability across the Paleozoic (Beaty & Planavsky, 2021; Dzombak & Sheldon, 2022). This stability likely reflects a balance between enhanced mineral dissolution driven by plant activity and increased nutrient and cation retention within thicker, biologically stabilized soils (Jobbágy & Jackson, 2001; Horodyskyj et al., 2012).

In addition, recent work suggests that terrestrial surfaces prior to and during early vascular plant colonization were not biologically inert. The widespread presence of microbial and cyanobacterial mats may have maintained relatively high baseline weathering intensity even in pre-vascular-plant landscapes, thereby reducing the contrast between pre- and post-vegetation weathering regimes (Finke et al., 2019; Beaty & Planavsky, 2021). Although the rise and diversification of vascular plants is associated with increased heterogeneity in weathering indices and subtle increases in Al mobility, these changes are expressed primarily as greater variability rather than a uniform global shift toward higher CIA values (Dzombak & Sheldon, 2022). We have discussed this in the revision on lines 96-101.

In the revision, we acknowledge that non-temperature factors such as soil calcification can influence CIA values, particularly in Phanerozoic paleosols where secondary carbonate accumulation is common. Because carbonate addition can artificially lower CIA, we explicitly screened the dataset to remove carbonate-rich samples following the approach of Lipp et al. (2021), thereby minimizing this bias (lines 602-6607).

3. Data Cleaning and Selection:

The manuscript lacks a detailed description of the data cleaning process. Specifically, how were issues such as K-metasomatism (A-CN-K trends), and the effects of aridity (e.g., during the Triassic) addressed?

R: Thank you for this comment, and apologies for the lack of detail here. We have expanded and clarified the data-cleaning and selection procedures in the revised manuscript. The workflow consists of the following steps (lines 589-647):

1.Initial filtering of the database. We first filtered the SGP dataset to retain only Phanerozoic siliciclastic samples and excluded records lacking major-element compositions, age constraints, or geographic coordinates.

2.Lithological screening. To minimize the effects of grain size and hydraulic sorting, we retained only fine-grained siliciclastic lithologies, including argillite, claystone, clay, meta-argillite, metapelite, mud, mudstone, silt, siltstone, metasiltstone, pelite, shale, slate, oil shale, and siltite.

3. Carbonate contamination screening. Because carbonate addition can artificially lower CIA values and produce unrealistically low temperature estimates, we removed samples with CaO contents greater than 8 wt% follow methods in Lipp et al., (2021) and Millikin et al., (2025).

4. Exclusion of documented recycled rocks and statistical outliers. To ensure CIA values reflect contemporaneous primary silicate weathering, we excluded samples explicitly described in their original publications as recycled or derived from pre-weathered sedimentary sources (e.g., Dumoulin and White, 2005; Young, 2004; Campbell et al., 2015). We further excluded extreme CIA outliers beyond 1.5× the interquartile range (IQR; i.e., below Q1 − 1.5×IQR or above Q3 + 1.5×IQR) to minimize the influence of sporadic anomalous samples that likely affected by sedimentary recycling or other post-depositional overprints.

5. K-metasomatism correction. To address diagenetic K enrichment, we applied established K-metasomatism corrections following Fedo et al. (1995), implemented using the framework discussed by Panahi et al., (2000) and Algeo et al. (2025). These corrections reduce the influence of post-depositional K addition on CIA values. Corrected CIA values are interpreted as upper-bound estimates, while uncorrected values represent lower-bound cases.

For the Triassic, CIA-derived GMST estimates are influenced primarily by diagenetic K enrichment. Application of the K-metasomatism correction substantially reduces downward bias in CIA values and improves agreement with independent reconstructions. Incorporation of the expanded dataset further enhances spatial representation. After these adjustments, Triassic GMST estimates indicate relatively high temperatures, with a central 50% range of approximately 15–30 °C, consistent with independent proxy evidence. Although regional aridity during parts of the Triassic could suppress chemical weathering intensity and locally bias CIA-derived temperatures, the Triassic warming trends after K-correction and sampling expansion suggests that aridity does not dominate the reconstructed global signal.

4. Model Extrapolation and CO_2 Forcing:
While I am not a specialist in the model used here, I have a question regarding the simulated climate under different CO_2 levels. I am particularly curious about the physical mechanism behind the apparent inverse relationship between temperature and the latitudinal temperature gradient at high CO2 concentrations (in Figure S9). Additionally, it would be very helpful if the CO_2 values used for each stage could be indicated directly in the relevant figures. Correlating these chosen values with CO_2 levels reconstructed by other methods would further strengthen the justification for the parameter selection.

R: We regret the lack of information on the occurrence of unusual inverted latitudinal temperature gradients at very high CO_2 levels. To explore very high pCO_2 conditions (up to 256× pre-industrial), we extrapolate from explicit GCM simulations at 1×, 2×, and 4× pCO_2 by scaling the zonal-mean temperature response approximately linearly with $\ln(pCO_2)$ given the known logarithmic relationship between CO_2 and global temperature. This approach yields inverted latitudinal temperature gradients under extreme greenhouse forcing, a behavior that has also been documented in recent oxygen isotope–model assimilation studies (Judd et al., 2024).

Physically, weak polar temperature inversions are dynamically feasible when polar regions are ocean-dominated and lack extensive landmasses. Differences in heat

capacity between land and ocean, the freezing point of seawater, and strong polar amplification can cause high-latitude temperatures to locally approach or slightly exceed those of adjacent mid-latitude land regions. At very high CO_2 levels, climate sensitivity and feedbacks are expected to become increasingly nonlinear, which may further exaggerate high-latitude warming relative to mid-latitudes and amplify these weak inversions. We discussed this on lines 754-764.

However, in our dataset, fewer than 3% of samples (497 records) are located at high latitudes (|latitude| > 65°), and the number of samples potentially affected by inverted gradients at extreme CO_2 levels is even smaller. To mitigate the influence of these rare cases, we applied two complementary strategies. First, we introduced a scale-factor adjustment based on a parabolic latitudinal temperature gradient (lines 777-782; Figs. S11-S13) which mitigates the occurrence of inverted temperature gradients. Second, we explicitly flattened inverted temperature gradients in sensitivity tests (lines 789-796; Figs. S14 and S15). We conclude that the presence of weakly inverted gradients occurs in a small number of the GCM fields, and does little to our overall reconstruction – not affecting our conclusions.

Minor Comments:

Line 50: multicellular organisms in the Cambrian-Ordovician “,” the colonization of land by plants in the Ordovician-Devonian

R: Added the comma between the two sentences.

Lines 95–97: Expand on how the expansion of vascular plants is reflected in the weathering intensity and aluminum mobility records.

R: We have modified this text in line with the response on this above. Lines 96-101.

Lines 122–129: Address potential latitudinal biases, especially given the Cenozoic data's reliance on North America. Can additional regional data be incorporated?

R: In the revision we have incorporated additional Cenozoic regional data, and have expanded our discussion on latitudinal bias. See lines 156-167 and Figure S5.

Line 137: Provide a detailed description of the data cleaning methodology.

R: As above, we have now included this in the manuscript. Lines 589-647.

Lines 198–202: Explain the higher GMST values during the P-C derived from CIA compared to oxygen isotopes (in figure2). If aridity is invoked for the Triassic, what explains the P–C discrepancy?

R: Thank you for raising this point. The elevated CIA-derived GMST values during parts of the Permian primarily reflected the inclusion of fine-grained sediments that have been explicitly documented as recycled or derived from pre-weathered sedimentary sources (e.g., Dumoulin and White; Young et al., 2004; Campbell et al., 2015). Such materials inherit prior weathering signatures and therefore do not necessarily record contemporaneous climatic conditions. In the revised manuscript, we have excluded all explicitly documented recycled samples to ensure that CIA values represent primary silicate weathering. Following this more conservative screening, alongside our other screening protocols and expanded dataset, the Permo-Carboniferous GMST estimates in the revised paper are now lower than for the Devonian and Triassic, in line with the glacial record (Fig. 3a).

For the Triassic, application of the K-metasomatism correction and incorporation of the expanded dataset means that Triassic GMST estimates indicate relatively high temperatures, consistent with lack of glaciation. Although regional aridity during parts of the Triassic could suppress chemical weathering intensity and locally bias CIA-derived temperatures, our predicted Triassic warming suggests that aridity does not dominate the reconstructed global signal here. We have updated the manuscript to note this.

Lines 611–619: Consider displaying the CO_2 values used in each stage and compare them with other published reconstructions.

R: Thanks for the suggestion. We've displayed the CO_2 value ranges predicted by this method for each stage and compared to proxy CO_2 reconstructions in Figure S7. We find the higher confidence range to be close to proxy records. Lines 731-736.

Lines 768–769: Discuss the implications of A-CN-K trends indicating potassium

metasomatism. How was diagenetic alteration accounted for, particularly given that K-metasomatism may depress CIA values and lead to underestimated temperatures?

R: As above, we agree that pervasive diagenesis, particularly K-metasomatism, can depress CIA values and bias reconstructed temperatures toward unrealistically low values. We have applied a correction for this in the revision. Lines 614-647.

Figure S9: Explain the occurrence of inverted latitudinal temperature gradients between 269–315 Ma, particularly starting at 4–8×CO_2.

R: As above, we have added a section on this. Lines 754-796. Figures S11-S15.

Reviewer #3 (Remarks to the Author):

I like this paper a lot. It’s great to have estimates of Phanerozoic temperatures independent of oxygen isotopes, especially those based on a well-established method like CIA. As I read the paper, I had many questions, but all were answered by the methods. The extensive methods are well described and seemingly address all caveats that I could think of. My comments are minor.

R: We thank the reviewer for these positive and encouraging comments.

There are a few grammatical issues, mostly missing commas, that should be cleaned up. I pointed some of them out.

R: We have carefully reviewed the manuscript, corrected the missing commas and other minor grammatical errors highlighted by the reviewer.

Line 50: comma needed.

Added.

Line 111: comma needed.

Added.

Line 60: I’ve always found these temperature estimates to be dubious.

R: These concerns are the main motivation for our study to develop an independent approach.

Line 144 through 148: This sentence needs to be two—too much information for 1.

Modified.

Line 149: comma needed.

Added.

Line 164: comma needed.

Added.

Lines 199 through 202: I see what you are saying about the Triassic, but I also think the Permian should be mentioned as a potential outlier. Your Permian estimate is below the other curves and the general trend of lower temperatures in Permian and warmer in the Triassic appears to be reversed in your dataset. Why? Even at the rough resolution of your dataset, doesn't that seem like a trend that would be preserved? Is it sampling? Are the bins biased by an overabundance of P-T boundary data?

R: As above, the elevated CIA-derived GMST values during parts of the Permian primarily reflect the inclusion of recycled sedimentary sources (e.g., Dumoulin and White; Young et al., 2004; Campbell et al., 2015). After the incorporation of the expanded dataset in the revision, together with stricter data cleaning (including removing documented recycled sources) and K-metasomatism correction, the Permian and Triassic GMST estimates now follow the trend suggested by the glacial record (Fig. 3a).

Equation 1: There is an extra space in CaO*

Modified.

Equation 2: I think there are missing parentheses.

Added.

Line 590: Shouldn't there be a last name to go with citation number 69? If not, shouldn't the number be non-superscript and separated from the preceding word? There are several others like that, and they should all be changed for readability.

Modified.

Figure S4 – 5: It would be more convenient to put the last name of the model author in the figure caption instead of a number.

Modified.

Figure S15 – 20: I like these figures, but the individual maps are way too small to be properly assessed. They all need to be bigger.

Modified.

References

- Algeo, T. J., Hong, H., & Wang, C. (2025). The chemical index of alteration (CIA) and interpretation of ACNK diagrams. *Chemical Geology*, *671*, 122474.
- Fedo, C. M., Wayne Nesbitt, H., & Young, G. M. (1995). Unraveling the effects of potassium metasomatism in sedimentary rocks and paleosols, with implications for paleoweathering conditions and provenance. *Geology*, *23*(10), 921-924.
- Panahi, A., Young, G. M., & Rainbird, R. H. (2000). Behavior of major and trace elements (including REE) during Paleoproterozoic pedogenesis and diagenetic alteration of an Archean granite near Ville Marie, Québec, Canada. *Geochimica et Cosmochimica Acta*, *64*(13), 2199-2220.
- Rudnick, D. T., Ortner, P. B., Browder, J. A., & Davis, S. M. (2005). A conceptual ecological model of Florida Bay. Wetlands, 25(4), 870-883. Farrell, Ú. C., Olson, H. C., Thompson, M. O., Abshire, M. L., Adeboye, O. O., Ahm, A. S. C., ... & Whiteside, J. H. (2025). The sedimentary geochemistry and paleoenvironments project phase 2 data release: An open data resource for the study of Earth's environmental history. Chemical Geology, 123148.
- Lipp, A. G., Roberts, G. G., Whittaker, A. C., Gowing, C. J., & Fernandes, V. M. (2021). Source region geochemistry from unmixing downstream sedimentary elemental compositions. Geochemistry, Geophysics, Geosystems, 22(10), e2021GC009838.
- Millikin, A. E., Gibson, T. M., Strauss, J. V., Bergmann, K. D., Tosca, N. J., Anderson, R. P., ... & Rooney, A. D. (2025). Geochemistry and mineralogy of Neoproterozoic strata in northeastern Svalbard: Reevaluating the prevalence of basalt weathering during the early Neoproterozoic. *Geological Society of America Bulletin*.
- Westerhold, T., Marwan, N., Drury, A. J., Liebrand, D., Agnini, C., Anagnostou, E., ... & Zachos, J. C. (2020). An astronomically dated record of Earth’s climate and its predictability over the last 66 million years. science, 369(6509), 1383-1387.
- Cenozoic CO2 Proxy Integration Project (CenCO2PIP) Consortium*†, Hönisch, B., Royer, D. L., Breecker, D. O., Polissar, P. J., Bowen, G. J., ... & Zhang, L. (2023). Toward a Cenozoic history of atmospheric CO2. *Science*, *382*(6675), eadi5177.
- Berner, R. A. (1997). The rise of plants and their effect on weathering and atmospheric CO2. *Science*, *276*(5312), 544-546.

- Beaty, B. J., & Planavsky, N. J. (2021). A 3 by record of a biotic influence on terrestrial weathering. *Geology*, *49*(4), 407-411.
- Dzombak, R. M., & Sheldon, N. D. (2022). Terrestrial records of weathering indicate three billion years of dynamic equilibrium. *Gondwana Research*, *109*, 376-393.
- Jobbagy, E. G., & Jackson, R. B. (2001). The distribution of soil nutrients with depth: global patterns and the imprint of plants. *Biogeochemistry*, *53*(1), 51-77.
- Horodyskyj, L. B., White, T. S., & Kump, L. R. (2012). Substantial biologically mediated phosphorus depletion from the surface of a Middle Cambrian paleosol. *Geology*, *40*(6), 503-506.
- Finke, N., Simister, R. L., O'Neil, A. H., Nomosatryo, S., Henny, C., MacLean, L. C., ... & Crowe, S. A. (2019). Mesophilic microorganisms build terrestrial mats analogous to Precambrian microbial jungles. *Nature Communications*, *10*(1), 4323.
- Lipp, A. G., Roberts, G. G., Whittaker, A. C., Gowing, C. J., & Fernandes, V. M. (2021). Source region geochemistry from unmixing downstream sedimentary elemental compositions. *Geochemistry, Geophysics, Geosystems*, *22*(10), e2021GC009838.
- Dumoulin, J. A., & White, T. (2005). *Micromorphologic evidence for paleosol development in the Endicott group, Siksikpuk formation, Kingak (?) shale, and Ipewik formation, western Brooks range, Alaska*. US Geological Survey.
- Young, L. E. (2004). A geologic framework for mineralization in the western Brooks Range, Alaska. *Economic Geology*, *99*(7), 1281-1306.
- Campbell, M., Rosenbaum, G., Shaanan, U., Fielding, C. R., & Allen, C. (2015). The tectonic significance of lower Permian successions in the Texas Orocline (Eastern Australia). Australian Journal of Earth Sciences, 62(7), 789-806.
- Judd, E. J., Tierney, J. E., Lunt, D. J., Montañez, I. P., Huber, B. T., Wing, S. L., & Valdes, P. J. (2024). A 485-million-year history of Earth's surface temperature. *Science*, *385*(6715), eadk3705.

Responses to reviewers:

Tight regulation of Earth's long-term temperature over Phanerozoic time

Dongyu Zheng, Alex G. Lipp, Alexander Farnsworth, Shufeng Li, Andrew S. Merdith, Khushboo Gurung, Mingcai Hou, Anqing Chen, Zixi Hou, Daniel J. Lunt, Erik A. Sperling, Paul J. Valdes, Benjamin J. W. Mills

The reviewers' comments are in black, and our responses in blue. Line numbers refer to the clean version of the revised manuscript.

Reviewer #1 (Remarks to the Author):

I am pleased to see that the authors have conducted a thorough revision; however, there are several critical issues I would like to further discuss:

While the sample size has increased significantly, in deep-time (especially Paleozoic) studies, sample quantity does not equate to spatial representativeness. I suggest that the authors provide a paleogeographic distribution map of the data points to demonstrate that the expanded dataset offers sufficient coverage across different paleolatitudes and tectonic blocks, rather than being a mere mechanical increase in data volume.

R: We agree that the paleogeographic coverage of samples is important. In the revised manuscript, we demonstrate that the expanded SGP2 dataset combined with the Cenozoic compilation provides broad paleogeographic coverage across the Phanerozoic. As shown in Figure 1b and Figures S21-S27, the samples are distributed across a wide range of paleolatitudes rather than being concentrated within a limited geographic area at the Period level. These maps illustrate that the expanded dataset improves both the number of samples and their spatial distribution, improving the robustness of the GMST reconstruction. We have clarified this point in lines 138-140, 159-162, and 603-611 of the revised manuscript.

The CIA-temperature conversion equation may exhibit a non-linear response under extreme cold or hot conditions. For instance, in hyper-greenhouse climates, CIA values may exceed 90, leading to a "saturation effect" (where the proxy becomes insensitive to further temperature increases). Conversely, in extreme icehouse conditions, physical

erosion dominates, and the chemical weathering signal might be completely obscured. I recommend that the authors briefly discuss the applicability of the equation in these end-member states or acknowledge the potential for larger non-linear errors at temperature extremes, while emphasizing the reliability of the results in capturing first-order patterns.

R: This is an important point. In our dataset, however, the potential impact of CIA saturation is limited. Only 206 samples (~1% of the total dataset) have CIA values greater than 90. Moreover, none of the early Paleozoic samples exceed this threshold, suggesting that the saturation effect is unlikely to significantly influence the reconstructed temperature trends for that interval. Therefore, the influence of CIA saturation on our GMST reconstruction is expected to be minor.

Nevertheless, we agree that non-linear relationships between weathering intensity and temperature may exist under extreme climatic conditions. To acknowledge this limitation, we have added further discussions in lines 145-148 and 721-727.

The authors utilize the average Upper Continental Crust (UCC) as a protolith proxy for potassium correction. Given the immense geological timescale of this study, variations in UCC composition over different periods could lead to substantial calculation biases. I suggest that the authors conduct a Sensitivity Analysis to verify whether fluctuations in parent rock composition might compromise the stability of the reconstructed Phanerozoic first-order temperature evolution trends.

R: This is a valuable suggestion. Although the average composition of the UCC is generally considered to have remained relatively stable through the Phanerozoic (Hawkesworth et al., 2020), estimates of UCC composition vary among different compilations and rock types. Because the potassium correction applied to CIA values depends on the parent-material molar K fraction, such variations could potentially influence the reconstructed temperatures.

To evaluate this effect, we conducted a sensitivity analysis using several representative UCC compositions. First, we adopted the average UCC composition reported by La Maitre et al. (2005) (Al_2O_3 = 14.6 wt%, Na_2O = 3.98 wt%, CaO = 4.32 wt%, K_2O =

2.95 wt%), which yields a parent-material molar K fraction of ~0.10 (0.103), consistent with estimates from Rudnick et al. (2005). We also considered the Paleozoic UCC composition proposed by Gaschnig et al. (2016) (Al_2O_3 = 11.2 wt%, CaO = 4.8 wt%, Na_2O = 1.6 wt%, K_2O = 2.4 wt%), which produces a higher K fraction of 0.136. In addition, we calculated the K fraction using a weathered young UCC composition from Kamber et al. (2005) (Al_2O_3 = 18.24 wt%, Na_2O = 0.79 wt%, CaO = 1.12 wt%, K_2O = 1.17 wt%), which yields a lower K fraction of ~0.08 due to its higher Al_2O_3 content and reduced Na_2O, CaO, and K_2O. These compositions therefore define a reasonable range of parent-material K fractions (~0.08–0.14). We recalculated CIA values and reconstructed GMST using these end-member scenarios. Although the absolute GMST values change slightly, the overall Phanerozoic warming and cooling trends remain essentially unchanged (Figs. S4 and S5). This result indicates that plausible variations in parent-rock composition have only a limited influence on the reconstructed first-order GMST evolution.

We have added this sensitivity analysis and discussion in lines 673-690 of the revised manuscript.

Regarding the grain-size effect, the authors' mention of "selecting fine-grained siliciclastic rocks" remains a qualitative treatment. In deep-time records, it is inherently difficult to completely exclude the dilution effect of silt on the clay fraction. The authors should, at the very least, verify whether the data sources include marine strata, considering the potential presence of authigenic clays formed through processes such as reverse weathering in marine environments. Furthermore, is it possible to utilize geochemical indicators (e.g., elemental ratios) or statistical methods to quantitatively evaluate the influence of sedimentary processes, such as grain-size sorting, on the weathering signals?

R: We agree that it is difficult to fully exclude the potential influence of authigenic clay formation associated with reverse weathering in marine environments. Because mineralogical and geochemical proxies required to quantify reverse weathering are not available in the SGP dataset, and depositional settings (marine versus terrestrial) are not consistently reported, a direct assessment of this process is not feasible. To partially evaluate its potential influence, we conducted a sensitivity analysis based on

depositional setting. Specifically, we reconstructed GMST using a subset of samples interpreted as terrestrial based on paleogeographic reconstructions. In this approach, a sample is considered terrestrial only if the surrounding 3 × 3 grid cells (approximately 11.25° longitude × 7.5° latitude) are all classified as land in the Scotese paleogeographic maps, providing a more conservative filtering criterion that minimizes inclusion of nearshore or uncertain environments. The GMST reconstruction based on this terrestrial-only subset shows broadly similar long-term trends compared to the full dataset, although some differences are observed, for example slightly lower temperatures during the Carboniferous that are more consistent with glacial conditions. These results suggest that the influence of marine authigenic clay formation is unlikely to dominate the first-order temperature signal, although some uncertainty may remain. We acknowledge that a more rigorous quantification of reverse weathering effects is difficult with the current dataset and may require additional constraints (e.g., mineralogical or geochemical indicators). This represents an important avenue for future work. We have added the sensitivity test in lines 883-902 and Figure S20, and included additional discussion of the limitations and potential directions for future work in lines 903-914.

Grain-size variations may also influence CIA values, as coarse-grained (e.g., sandy) sediments typically exhibit lower CIA values than fine-grained, clay-rich sediments due to dilution by quartz and feldspar (Galy and France-Lanord, 2001; Lupker et al., 2013). In the SGP and Cenozoic data compilation, we primarily selected silty- to clay-sized siliciclastic rocks to reduce potential grain-size effects. To further quantitatively evaluate this influence, we used the Al_2O_3/SiO_2 ratio as a grain-size proxy (Guo et al., 2018), where clay-rich sediments generally exhibit higher ratios, whereas quartz-rich, coarser sediments show lower values. The temporal distribution of Al_2O_3/SiO_2 across the Phanerozoic shows no systematic long-term trend, suggesting that the dataset is not strongly biased toward particular grain-size fractions through time. In addition, the correlation between CIA and Al_2O_3/SiO_2 is relatively weak ($r = 0.33$), indicating that grain-size sorting is unlikely to be the primary control on CIA values. To further assess this effect, we applied a conservative filter by excluding samples with $Al_2O_3/SiO_2 < 0.1$, which likely represent quartz-rich, coarse-grained sediments. The GMST reconstruction based on this filtered dataset shows nearly identical warming and cooling patterns to those presented in Figure 3a. This comparison suggests that grain-size

effects do not exert a strong influence on the reconstructed first-order GMST trends. We have added this discussion in lines 866–882. See Figures S18 and S19.

Reviewer #2 (Remarks to the Author):

I have carefully read the authors' response and the revised manuscript. I believe the authors have addressed the reviewers' comments and provided a thorough response, particularly regarding their efforts to evaluate the local signals of the CIA and the effects of K-metasomatism during data cleaning and processing.This study presents a novel alternative method for paleotemperature reconstruction using big data and is worthy of publication.

R: We thank the reviewer for constructive comments!

References

- Hawkesworth, C.J., Cawood, P.A. and Dhuime, B., 2020. The evolution of the continental crust and the onset of plate tectonics. Frontiers in earth science, 8, p.326.
- Le Maitre, R.W., Streckeisen, A., Zanettin, B., Le Bas, M.J., Bonin, B. and Bateman, P. eds., 2005. Igneous rocks: a classification and glossary of terms: recommendations of the International Union of Geological Sciences Subcommission on the Systematics of Igneous Rocks. Cambridge University Press.
- Rudnick, R. L., Holland, H. D. & Rudnick, R. L. The crust. vol. 3 (Elsevier, 2005).
- Gaschnig, R.M., Rudnick, R.L., McDonough, W.F., Kaufman, A.J., Valley, J.W., Hu, Z., Gao, S. and Beck, M.L., 2016. Compositional evolution of the upper continental crust through time, as constrained by ancient glacial diamictites. Geochimica et Cosmochimica Acta, 186, pp.316-343.
- Kamber, B.S., Greig, A. and Collerson, K.D., 2005. A new estimate for the composition of weathered young upper continental crust from alluvial sediments, Queensland, Australia. Geochimica et Cosmochimica Acta, 69(4), pp.1041-1058.

- Guo, Y., Yang, S., Su, N., Li, C., Yin, P. and Wang, Z., 2018. Revisiting the effects of hydrodynamic sorting and sedimentary recycling on chemical weathering indices. Geochimica et Cosmochimica Acta, 227, pp.48-63.
- Galy, A., & France-Lanord, C. (2001). Higher erosion rates in the Himalaya: Geochemical constraints on riverine fluxes. Geology, 29(1), 23-26.
- Lupker, M., France-Lanord, C., Galy, V., Lavé, J., & Kudrass, H. (2013). Increasing chemical weathering in the Himalayan system since the Last Glacial Maximum. Earth and Planetary Science Letters, 365, 243-252.

Responses to reviewers:

Tight regulation of Earth's long-term temperature over Phanerozoic time

Dongyu Zheng, Alex G. Lipp, Alexander Farnsworth, Shufeng Li, Andrew S. Merdith, Khushboo Gurung, Mingcai Hou, Anqing Chen, Zixi Hou, Daniel J. Lunt, Erik A. Sperling, Paul J. Valdes, Benjamin J. W. Mills

The reviewers' comments are in black, and our responses in blue. Line numbers refer to the clean version of the revised manuscript.

REVIEWERS' COMMENTS

Reviewer #1 (Remarks to the Author):

I am pleased with the authors' quick and comprehensive response to the feedback. The manuscript is now in excellent shape. I am confident that this work will contribute meaningfully to the study of deep-time environments and geochemistry. While many questions in this field warrant further discussion, there is clearly much more to be unraveled from the siliciclastic record.

We thank reviewer's constructive comments for our manuscript, which significantly improved the quality of this work.
